# Use of a shared decision-making intervention to support treatment decision-making for patients following an anterior cruciate ligament rupture: a mixed methods feasibility study

**DOI:** 10.1136/bmjopen-2024-095189

**Published:** 2025-08-27

**Authors:** Hayley Carter, David Beard, Charlotte Dodsley, Paul Leighton, Joshua McCallion, Fiona Moffatt, Benjamin Edward Smith, Kate E Webster, Phillipa Logan

**Affiliations:** 1 Physiotherapy Outpatients, Florence Nightingale Community Hospital, University Hospitals of Derby and Burton NHS Foundation Trust, Derby, UK; 2 University of Nottingham, School of Medicine, Nottingham, UK; 3 Surgical Intervention Trials Unit (SITU), Botnar Research Centre, University of Oxford NDORMS, Oxford, UK; 4 NHMRC Clinical Trials Centre, Faculty of Medicine and Health, University of Sydney AU, University of Sydney, Sydney, New South Wales, Australia; 5 Patient Representative, The POP-ACLR Study, Derby, UK; 6 University of Nottingham, School of Health Sciences, Nottingham, UK; 7 School of Allied Health, Human Services and Sport, La Trobe University, Melbourne, Victoria, Australia; 8 Surgical, Treatment and Rehabilitation Service (STARS) Education and Research Alliance, The University of Queensland, Brisbane, Queensland, Australia

**Keywords:** Orthopedics, Decision Making, Feasibility Studies

## Abstract

**Objectives:**

To understand feasibility, acceptability and indicators of effectiveness of a shared decision-making (SDM) intervention with patients following an anterior cruciate ligament (ACL) rupture.

**Design:**

Non-randomised feasibility study with embedded qualitative interviews.

**Setting:**

Orthopaedic and physiotherapy service at an acute National Health Service (NHS) Teaching Hospital in the Midlands, UK, between 29 January and 5 June 2024.

**Participants:**

Patients diagnosed with an ACL rupture following MRI.

**Intervention:**

Delivery of a SDM intervention which comprised of two components: (1) patient information leaflet and (2) option grid.

**Outcome measures:**

The primary outcome was to determine feasibility for a definitive trial using four outcomes: (1) recruitment rate, (2) fidelity of intervention delivery, (3) acceptability and (4) follow-up questionnaire completion. The secondary outcome was to explore indicators of the intervention’s effectiveness using quantitative data from patient reported outcome measures (acceptability questionnaire and satisfaction with decision scale) and qualitative data from patient and clinician interviews.

**Results:**

21 patients were approached to take part in the study, 20 were recruited with a mean age of 32.2 (SD 9.7), 40% were female. The recruitment rate was 95.2%, fidelity of intervention 100%, acceptability 94% and follow-up questionnaire completion 100%. The mean overall satisfaction with decision scale score was 24.85/30 (SD 3.82). There were no adverse events. Data from qualitative interviews with patients (n=5) and physiotherapists (n=5) suggested the SDM was acceptable and appeared effective in: (1) supporting decision-making about treatment, (2) conversations between patients and clinicians, (3) improving patient knowledge, (4) providing patients with access to health language and (5) supporting patients to ask questions deemed important to them.

**Conclusion:**

The novel SDM intervention is acceptable to both patients and physiotherapists. Indicators of effectiveness explored through quantitative and qualitative data suggest the intervention to be beneficial to decision-making processes for patients and clinicians deciding on treatment following an ACL rupture. All four feasibility outcomes were achieved, indicating a full trial is feasible to run in the NHS.

**Trial registration number:**

ISRCTN17801081.

**STRENGTHS AND LIMITATIONS OF THIS STUDY**

- This feasibility study successfully used a mixed-methods approach to understand feasibility, acceptability and indicators of effectiveness of the novel shared decision-making intervention.
- Qualitative interviews supported deeper exploration among patients and physiotherapists in addition to providing further context to quantitative data.
- This was a single site study and so feasibility and individual experiences at other National Health Service trusts may differ and warrant further exploration.

## Introduction

 Anterior cruciate ligament (ACL) ruptures affect over 20 000 individuals in the UK each year.^1 2^ Three randomised controlled trials have compared surgical and non-surgical management for patients following acute and non-acute ACL rupture and report conflicting findings.^3^,^5^

Given the uncertainty of the evidence base, it is not unexpected that patients also report confusion and ambiguity regarding decision-making about their choice of treatment.^6^ A UK qualitative study (n=18) exploring the experiences of patients on the surgical pathway found patients to describe the decision-making process in three ways. First, some participants reported to have felt the decision about treatment was made for them with limited opportunity to be involved in the decision-making process. Second, other participants reported a desire to avoid responsibility for deciding on treatment and preferred to defer responsibility to an experienced clinician. Finally, participants described being presented with unbalanced information regarding treatment and felt there was no decision to be made as advice favoured surgical intervention to support a return to physical activity/sport. This left them with little choice but to opt for surgery.^6^ Decision-making in healthcare is multifactorial and a patient’s motivation to be involved in the process is complex. For other orthopaedic conditions, such as knee osteoarthritis, decision-making interventions (such as Option Grids) have shown success in supporting patients to make decisions about the management of their condition.^7^

A 2023 nominal group consensus study co-produced a shared decision-making (SDM) intervention with patients and key stakeholders with the aim of supporting treatment decisions for patients following an ACL rupture. The development process was underpinned by the Extended Normalisation Process Theory (ENPT) to ensure factors concerning implementation of the intervention in clinical practice were considered and embedded within the design.^5^ The co-production process, underpinned by relevant theory, ensured the intervention was based on the latest available evidence alongside patient experiences and expert opinion, following Medical Research Council guidance for complex intervention development.^8^ The development process is described in further detail in a separate publication.^9^ The intervention, comprising a patient information leaflet and option grid, aims to ensure patients can make informed decisions about their treatment and that, if chosen, the surgical pathway is appropriate. The purpose of this study was to explore feasibility, acceptability and indicators of effectiveness of this novel SDM intervention ahead of a potential future main trial.

## Methods

This study was reported in accordance with the Consolidated Standards of Reporting Trials (CONSORT) statement: extension to randomised pilot and feasibility trials in addition to published guidance of its use in non-randomised studies.^10 11^ The CONSORT checklist is available in online supplemental file 1. As the study encompassed a qualitative component, the Standard for Reporting Qualitative Research (SRQR) was also used. The SRQR checklist is available in online supplemental file 2.

This study was registered with ISRCTN (ISRCTN17801081).

A full description of the methods has previously been published.^12^ A brief description is detailed below. There were no changes or deviations from the published protocol.

### Study aim

The objective of this study was to understand (1) feasibility, (2) acceptability and (3) indicators of effectiveness, of an SDM intervention with patients following an ACL rupture in a UK National Health Service (NHS) setting.

### Design

A non-randomised feasibility study with embedded qualitative interviews.

### Sample size and recruitment

Participants were recruited between 29 January 2024 and 5 June 2024 from the orthopaedic and/or outpatient musculoskeletal physiotherapy services at one large acute NHS teaching hospital trust (University Hospitals of Derby and Burton NHS Foundation Trust,UHDB), across three hospitals. Clinical staff also identified appropriate patients from orthopaedic and physiotherapy clinic/waiting lists. They confirmed eligibility (shown in box 1) and gained consent from the researcher to make contact with the patient to discuss the study. On confirmation that the potential participant met the eligibility criteria, they were invited to participate in the study and provided with the participant information sheet and consent documents.

Box 1 **Eligibility criteria**

**Inclusion criteria**

- Age 18 or over.
- First-time anterior cruciate ligament (ACL) rupture (in that limb) confirmed by an MRI scan.

**Exclusion criteria**

- Concomitant injury requiring surgical intervention anticipated to significantly alter usual treatment, for example, fracture, bucket handle meniscal tear requiring immediate surgical intervention prior to ACL reconstruction (ACLR).
- Previous surgery to the affected limb.
- Pregnancy (as this is likely to affect decision-making regarding surgical treatment and rehabilitation).

We aimed to recruit 20 participants with a maximum 6-month recruitment window.^9^ We planned to interview a purposive sample of six patients (aiming for variability in age, sex, education and health literacy level, response to baseline and follow-up questionnaires) and six physiotherapists (aiming for variability in level of experience, hospital site, number of participants treated in the trial).^10^

### Data collection

Once consented, baseline data were collected on patient demographics, mechanism of injury, pre-injury and current physical activity level/frequency and received treatment recommendation. Participants’ postcodes were used to calculate the Indices of Multiple Deprivation 2019 (IMD2019) decile which represents 10 equal sized groups from 1 (most deprived 10% of areas nationally) to 10 (least deprived 10% of areas nationally).^13^ The Rapid Estimate of Adult Literacy in Medicine, Revised (REALM-R) was also recorded to identify those at risk of poor literacy skills.^14^

Participants were given the choice of receiving the follow-up questionnaires (satisfaction with decision (SWD) and acceptability questionnaire) online (via email and/or text message) or via paper. Reminders were sent once a week (via the preferred method) for three further weeks until the questionnaires were completed.

### Intervention

#### Shared decision-making intervention

The SDM intervention, available in online supplemental file 3, comprised of two components:

1. Patient information leaflet
2. Option grid

The patient information leaflet was a 10-page booklet including information on: knee anatomy, treatment options, outcomes and risks, pathway timelines, key questions for patients to consider and a blank notes section. The option grid was a two-sided A4 page directly comparing surgical with non-surgical treatment in response to 11 key questions (shown in online supplemental file 3). Two additional questions prompted patients to consider and document clinician recommendations about treatment and their treatment preferences. The two components were combined to form the single intervention. The intervention has been described in greater detail elsewhere.^9^

Participants were able to access the intervention via a physical paper copy and/or digitally via pdf documents. The paper and/or digital intervention was provided to patients on consenting to take part in the feasibility study (according to participant preference). The digital copy was delivered via the study website (www.pop-aclr.co.uk) on a password protected page allowing patients to download pdf versions of the paper documents. The intervention was subsequently discussed with patients at an outpatient appointment with a trained physiotherapist.

#### Training of the physiotherapists

The training consisted of a 1 hour online or in person session and included approximately 30 minutes of content on study background, intervention development process, trial overview, schedule of events and participant flow and outcome measure data collection. The remaining 30 minutes was used for intervention training covering its components and delivery method in practice.

Following training, physiotherapists were provided with a one-sided information document summarising key points of the study and access to a restricted page on the study website. The restricted page included: key study information, downloadable versions of the protocol, case report form, intervention and participant information sheets for patients in the feasibility study and physiotherapy interviews.

All physiotherapists were supported via email or informal conversation where required. A key contact (study champion) was identified at the two participating physiotherapy sites to support face-to-face discussion where needed. Individual physiotherapists also received a summary of study procedures via email ahead of the planned consultation with a patient participating in the study.

### Outcomes

The study collected outcomes relating to feasibility and indicators of intervention effectiveness.

#### Feasibility

There were four main feasibility outcomes: (1) recruitment rate, (2) fidelity, (3) acceptability and (4) follow-up questionnaire completion.

1. Recruitment rates were recorded and defined as the number of participants approached to participate in the study following confirmation of eligibility.
2. Fidelity was defined as adherence to the delivery of the intervention. This was evaluated using a case report form completed by the treating physiotherapist detailing the clinical consultation where the SDM intervention was discussed.
3. Acceptability was measured using both quantitative and qualitative data. All patients participating in the study were asked to complete an acceptability questionnaire after the scheduled consultation with the physiotherapist. Acceptability was further explored during individual qualitative interviews with both patients and physiotherapists.
4. Follow-up questionnaire completion was defined as the percentage of returned patient-reported outcomes (PROs) at 4 weeks. The percentage of missing data was also recorded.

Feasibility was also evaluated through qualitative interviews, retention rates and reasons for withdrawal.

#### Patient-reported outcomes

PROs, acceptability questionnaire and SWD scale, were collected up to 4 weeks following the SDM consultation.

The bespoke acceptability questionnaire, online supplemental file 4, was designed and included 15 statements pertaining to key elements thought to determine acceptability. These statements were discussed with participants of the consensus study where the intervention was developed. Feedback on the acceptability statements was therefore gained from clinicians, a therapy manager, patients and academic experts.

The SWD scale comprises six items scored on a 5-point Likert scale to determine a patient’s satisfaction with a healthcare decision.^15 16^ For each item, a level of satisfaction is reported from strongly agree (5) to strongly disagree (1) with an overall level of satisfaction reported between 5 (low satisfaction with decision) to 30 (high satisfaction with decision). The SWD scale is shown in online supplemental file 5.

### Nested qualitative interviews

A sample of five participants and five physiotherapists were interviewed. As described in the protocol, participants were interviewed until data saturation was reached. Semi-structured interviews were conducted using a topic guide (online supplemental file 6). Participants (patients and physiotherapists) were offered an interview at a time and via a medium convenient for them (in-person, telephone or Microsoft Teams). Interviews were conducted by the lead researcher (HC) who is also a physiotherapist working at UHDB.

**Figure 1:**
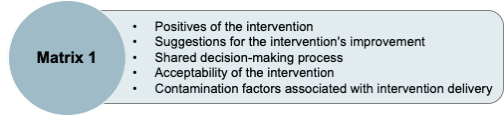
Data matrix guiding qualitative interview data collection and analysis.

The framework approach was used to analyse interview data which was mapped onto a matrix with five overarching headings shown in figure 1.

### Data analysis

Quantitative and qualitative data analysis were completed concurrently. Electronic quantitative study data were collected and managed using REDCap electronic data capture tools hosted at the University of Nottingham.^17^ Statistical analysis was undertaken using SPSS Statistics for Mac, V.29.0.1.0 (SPSS, Chicago, Illinois, USA). Qualitative data was stored and managed in NVivo V.14. Data were mapped onto predefined matrices in Microsoft Excel (Microsoft, Redmond, Washington, USA).

### Quantitative data

Descriptive statistics were used to summarise the baseline data. The continuous baseline variables (eg, age) were reported with means, SD and 95% CIs where appropriate, if shown to be normally distributed, otherwise were reported with medians and IQRs. The categorical variables (eg, sex) were reported with frequencies and percentages. Responses for participants from a low level of education, those at risk of poor health literacy and from more deprived areas were compared using an independent samples t-test (parametric assumptions met) and Mann-Whitney U test (parametric assumptions not met), with a significance level at p<0.05.

#### Qualitative data

Interviews were recorded and transcribed verbatim using an approved third-party transcription service (TP transcription, https://tptranscription.co.uk). All transcriptions were checked for accuracy against original recordings. The researcher also maintained a reflexive journal to document thoughts after each interview, on initial reading of the transcripts and during data analysis.

Data were mapped onto a matrix with five overarching headings (presented above, figure 1) by HC. Data were discussed with the study team during trial management meetings after charting was completed for 25% of the dataset. Data pertaining to implementation factors were mapped onto a second matrix underpinned by the ENPT (reported in a separate publication). Following mapping, data were then organised into two broad topics. Topic 1 (explored in this paper) included three themes derived from the five overarching headings in Matrix 1: acceptability of the intervention, indicators of effectiveness and trial procedures (including feasibility). Topic 2 (explored in a separate publication) reports implementation of the intervention and considerations for future implementation and normalisation.

### Patient and public involvement

Patients and key stakeholders (including orthopaedic surgeons, therapy managers and therapists) with experience of sustaining an ACL rupture, treating patients following rupture or managing departments where patients seek treatment following an ACL rupture were involved in the grant funding application, study design and set-up. This supported research decision-making regarding participant eligibility criteria, sample size for the study, the research methods and analytical approach.

## Results

A total of 20 participants were recruited into the study. Baseline data are shown in table 1.

**Table 1:** Summary of baseline data

Baseline data summary	
Age (years), mean (SD, range)	32.2 (9.7, 18–57)
Sex at birth, n (%)	
Female	8 (40)
Male	12 (60)
Gender, n (%)	
Identity the same as sex registered at birth	20 (100)
Ethnicity, n (%)	
White British	18 (90)
Asian Pakistani	1 (5)
British Indian	1 (5)
Index of Multiple Deprivation decile, n (%)*	
2	1 (5)
3	1 (5)
5	2 (10)
6	1 (5)
7	3 (15)
8	6 (30)
9	3 (15)
10	3 (15)
Highest level of education, n (%)†	
No formal qualifications	1 (5)
Level 2	4 (20)
Level 3	6 (30)
Level 4	1 (5)
Level 5	2 (10)
Level 6	6 (30)
REALM-R, n (%)‡	
3	1 (5)
5	1 (5)
6	1 (5)
7	7 (35)
8	10 (50)
Employment status, n (%)	
Full-time	16 (80)
Part-time	1 (5)
Full-time or part-time student	2 (10)
Full-time carer	1 (5)
Days between injury and diagnosis, median (range, IQR values)	38 (5–2191, 8–65.5)
Mechanism of injury, n (%)	
Contact	6 (30)
Non-contact	14 (70)
Activity engaged in at the time of injury, n (%)	
Football	13 (65)
Skiing	4 (20)
Hockey	1 (5)
Netball	1 (5)
Motorbike accident	1 (5)
Treatment recommendation, n (%)	
Surgery	12 (60)
Non-surgery (rehabilitation)	3 (15)
Provided with the option for surgical or non-surgical intervention and placed on the waiting list	5 (25)
Preinjury physical activity level, n (%)	
Competitive – national level	1 (5)
Competitive – regional level	3 (15)
Competitive – local level	9 (45)
Recreational/leisure	6 (30)
Occasional participation	1 (5)
Preinjury physical activity frequency, n (%)	
Once a week	1 (5)
2–4 times a week	11 (55)
5–7 times a week	7 (35)
More than 7 times a week	1 (5)
Current physical activity level, n (%)	
Recreational/leisure	2 (10)
Occasional participation	7 (35)
Unable to participate due to knee injury	11 (55)
Current physical activity frequency, n (%)	
Once a week	2 (10)
2–4 times a week	7 (35)
Unable to participate	11 (55)

* Index of Multiple Deprivation decile represents 10 equal sized groups from 1 (most deprived 10% of areas nationally) to 10 (least deprived 10% of areas nationally).

† Level of education is reported from no formal qualification to level 8, examples of qualifications at each level include: Level 1, GCSE grades D–G or 3–1; Level 2, GCSE grades A*–C or 9–4; Level 3, A level; Level 4, certificate of higher education; Level 5, diploma of higher education; Level 6, degree with honours; Level 7, master’s degree; Level 8, doctorate.

‡ REALM-R scores are reported from 0 to 8, a score of 6 or below identifies an individual to be at risk of poor health literacy.

REALM-R, Rapid Estimate of Adult Literacy in Medicine, Revised.

**Figure 2:**
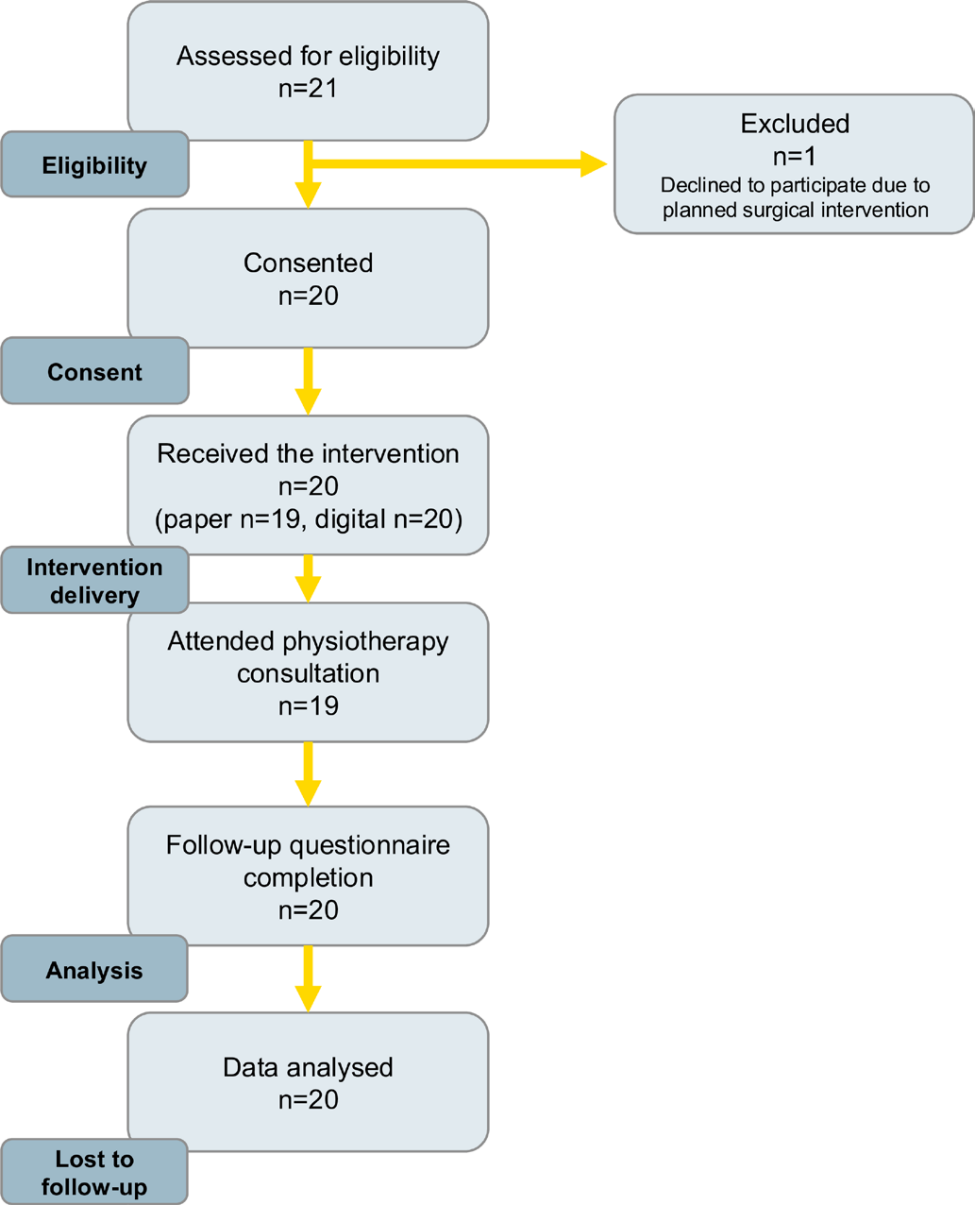
CONSORT (Consolidated Standards of Reporting Trials) flow diagram.

A CONSORT diagram is shown in figure 2.

### Feasibility outcomes

#### Recruitment rate

21 patients met the inclusion criteria and were approached about the study. 20 consented to take part, resulting in a recruitment rate of 95.2%. One participant declined to participate as, ahead of their MRI result, they had booked a provisional date to have an ACL reconstruction (ACLR) in the private sector.

#### Fidelity to the intervention

All physiotherapists completed the case report form in full and indicated 100% intervention fidelity. There were 11 physiotherapists who delivered the intervention.

#### Acceptability of the intervention

20 participants completed the acceptability questionnaire. Overall acceptability was 93.7%. Results for responses to each statement are shown in table 2.

**Table 2:** Acceptability questionnaire

Acceptability statement	Agree n (%)	Disagree n (%)	Level of agreement (%)
The information in the shared decision-making tools was helpful	19 (95)	1 (5)	95
The information helped me to understand my ACL tear	20 (100)	0 (0)	100
The information helped me to understand my treatment options	20 (100)	0 (0)	100
The diagram on page 8 of the information leaflet was helpful in getting me to think about key questions to discuss with my healthcare professional	19 (95)	1 (5)	95
The option grid helped me to make a decision	18 (90)	2 (10)	90
I am aware of the advantages of each treatment option for my ACL injury	20 (100)	0 (0)	100
I am aware of the disadvantages of each treatment option for my ACL injury	19 (95)	1 (5)	95
I understood all the information in the shared decision-making tools	20 (100)	0 (0)	100
The length of the shared decision-making tools was ‘just right’	19 (95)	1 (5)	95
The length of the shared decision-making tools was ‘too short’	3 (15)	17 (85)	85
The length of the shared decision-making tools was ‘too long’	3 (15)	17 (85)	85
The presentation of information seemed in favour of having surgery	4 (20)	16 (80)	80
The presentation of information seemed in favour of not having surgery	3 (15)	17 (85)	85
The presentation of information was ‘balanced’ and fair towards each treatment option	20 (100)	0 (0)	100
I would recommend the shared decision-making tools to other patients in a similar position to me	20 (100)	0 (0)	100
	Overall percentage agreement (%)		93.67

ACL, anterior cruciate ligament.

#### Follow-up questionnaire completion

All 20 participants completed both PROs (acceptability and SWD scale) resulting in a completion rate of 100%. All questionnaires were completed online via email or text message links.

### Outcome measures

#### Acceptability

The overall level of acceptability was 93.67% as reported above. Acceptability was also explored during individual qualitative interviews (discussed below).

#### Satisfaction with decision

The mean SWD score across all 20 participants was 24.85 out of 30 (95% CI 23.06 to 26.64, SD 3.82). The lowest score was 17, while the highest (reported by two participants) was the maximum score of 30. Only one participant disagreed with one statement (that they were adequately informed); all other responses were recorded as ‘neither agree nor disagree’, ‘agree’ or ‘strongly agree’.

#### Sensitivity analysis

To determine utility of the SDM intervention among those from (1) a lower socioeconomic background, (2) with a lower level of education and (3) at risk of poor health literacy, we performed a sensitivity analysis against these three variables.

#### Indices of Multiple Deprivation

Participants with an IMD of 5 or below (n=4, 20%) had an overall lower percentage agreement on the acceptability questionnaire (91.67%) and lower mean SWD score (24.5) than those with an IMD>5 (acceptability 94.17%, SWD 24.95). The differences, however, were not statistically significant (SWD p=0.422, acceptability p=0.820).

#### Rapid Estimate of Adult Literacy in Medicine, Revised

Three participants (15%) were identified to be at risk of poor health literacy, scoring 6 or below on REALM-R. Those at risk of poor health literacy had an overall lower percentage agreement on the acceptability questionnaire (91.11%) and lower mean SWD score (24.33) than those not at risk of poor health literacy (acceptability 94.12%, SWD 24.94). The differences were not statistically significant (SWD p=0.807, acceptability p=1.0).

#### Level of education

Over 50% of participants reported to hold a qualification at level 3 or below (n=11, 55%). Participants with a level 3 qualification or below had an overall higher percentage agreement on the acceptability questionnaire (91.11%) and lower mean SWD score (24.33) than those with a level 4 qualification or above (acceptability 92%, SWD 23.9). The differences were not statistically significant (SWD p=0.321, acceptability p=0.230).

### Nested qualitative interviews

Individual semi-structured interviews were completed with five patient participants and five physiotherapist participants. Demographic data of the patient and physiotherapist participants is shown in online supplemental file 7. Interview data are described in relation to three themes exploring: (1) acceptability of the intervention, (2) indicators of effectiveness, and (3) feedback of trial procedures.

#### Theme 1: acceptability of the intervention

The SDM intervention was positively received by patients and physiotherapists and overall deemed to be acceptable with respect to its content and delivery. Patients reflected positively on the intervention’s length, colour scheme, font size, level of detail, medical language and lay terminology used, paper quality and availability of the paper and online version. Participants suggested that the intervention struck an appropriate balance of offering relevant information without excessive, lengthy and confusing medical detail (Quote 1, online supplemental file 8). Two participants explained to be disappointed by some data as most expected the outcomes of surgery to be higher than that reported in the literature. However, they went on to reflect positively about their inclusion as it offered transparency of outcomes for the available treatment options (Quote 2, online supplemental file 8).

Both patients and physiotherapists reflected positively on the timing of intervention delivery, with patients highlighting the value of its provision at the point of diagnosis, followed by an appointment where it was expected to be discussed. Patients reported that this allowed time to process the information and consider relevant questions. All participants felt that the physiotherapist was an appropriate healthcare professional to be discussing decision-making about treatment with (Quote 3, online supplemental file 8). One patient reflected on the importance of the healthcare professional to be interested in the area and not ‘just going through the motions’ (0012, patient), with another commenting on their preference for delivery by a ‘knee specialist’ (0009, patient). One participant suggested a one-to-one appointment may not be necessary. They felt the SDM intervention contained sufficient detail to support decision-making about treatment, particularly where ACLR was seen to be inevitable for young patients looking to return to high-level cutting and pivoting sports.

Physiotherapists reported the intervention to be acceptable, describing it to be non-burdensome, compatible with usual practice, low risk and within the scope of a qualified physiotherapist’s skillset. The intervention was low/minimal cost to participants apart from the time taken to engage with it, which was reported to be acceptable. One therapist reflected on the increased time of a 60-min appointment allowing for more in-depth conversations, which was preferred to 30 min. In contrast, some physiotherapists felt the intervention supported a reduction in consultation time.

#### Theme 2: indicators of effectiveness

The SDM intervention was reported to add value in several areas of practice by both patients and physiotherapists. Patients described the intervention to increase their knowledge of the injury and the treatment options available to them which supported them to engage in decision-making (Quote 4, online supplemental file 8). In contrast, one participant reflected on the lack of compelling evidence available and reported in the SDM intervention and suggested this added difficulty to the decision-making process (Quote 5, online supplemental file 8). Inclusion of the statistical data was seen to add value. It was viewed as evidence-based and relevant to what happens in real life. Several patients commented on the use of statistics to support an individual assessment of the pros and cons of treatment, in line with what was important to them (Quote 6, online supplemental file 8). Physiotherapists also reported the level of detail, statistical data and information included in the intervention to be appropriate, relevant and useful. They reflected that, without the intervention, they would not have been able to offer the same level of evidence-based information as keeping up-to-date with the literature is challenging in the current NHS climate (Quote 7, online supplemental file 8).

The intervention was also described to support communication between patients and physiotherapists. Further, all patients reported to use the intervention outside of the trial, with friends, family members, healthcare professionals and employers to help communicate their injury and gain support to manage its consequences. Both physiotherapists and patients deemed the intervention to be trustworthy as it was derived from research and included references to the literature that were easy to locate should participants want to seek further information. As a result, some patients suggested to have not needed to conduct internet research to understand their injury further, saving them time from reading (or watching) unwanted levels of detail (Quote 8, online supplemental file 8). Physiotherapists further reported the intervention to have supported ‘buy in’ from patients, who were perceived to have increased knowledge of their injury and treatment options available to them. They felt the intervention offered a good foundation level of knowledge for patients which supported a more pragmatic appointment that was structured, streamlined and tailored to each individual (Quote 9, online supplemental file 8). This was also mirrored by patients who reported the interventions’ value in providing them with access to healthcare language. Patients reflected on their ‘non-medical background’ and explained that the intervention increased their understanding of appropriate terminology, allowing them to have individualised conversations with healthcare professionals and ask important questions relevant to them (Quote 10, online supplemental file 8).

In addition, physiotherapists described the role of the intervention in empowering patients to be involved in their care. They felt this enabled a greater sense of control and increased patients’ motivation as they were perceived to have higher accountability for the treatment and its outcomes. They reflected on the interventions presentation of information and felt it was fair and balanced towards both treatment options, ensuring it was effective for use with all patients following an ACL rupture. Physiotherapists further described the interventions utility in supporting their skills to deliver evidence-based information and have SDM conversations with patients. This was reflected by physiotherapists in relation to patients following an ACL rupture, but also for patients with other musculoskeletal/orthopaedic conditions as part of usual practice as opposed to randomised controlled trial (RCT) type practice. They explained to feel more confident to take part in SDM and explore patient views about treatment (Quote 11, online supplemental file 8).

#### Theme 3: trial procedures

Both patients and physiotherapists had a positive attitude towards participating in the feasibility study. Patients reflected on the low burden of engagement with the intervention, completing baseline data collection and PROs electronically in person or via mobile and email links. Three areas identified by physiotherapists for future trial refinement included, use of drop-in sessions for training refreshers (although not favoured by all), a QR code to access the clinician resources page on the website (replacing access via a password which was or could be forgotten) and a reminder/checklist of key intervention components to be embedded within a patient’s electronic record. Physiotherapists reported the use of a ‘study champion’ and availability to email and receive a prompt response from a member of the research team to have been successful during the feasibility study and should be incorporated into a future main trial to support delivery.

Future contamination factors were also explored with physiotherapists. When asked about the delivery of usual care in parallel to the SDM intervention (as part of an effectiveness trial), they expressed perceived difficulty with doing so given the new knowledge obtained from the SDM intervention. They favoured a design where treatment would be delivered for one intervention arm only. This is an important consideration and reflection given some therapists had also already begun using the intervention, or elements of it, in their usual practice.

### Shared decision-making intervention refinements

As a result of participant feedback during interviews, the following refinements were suggested: (1) additional information to be added about the use of braces for non-surgical management of ACL ruptures, and where to purchase from (2) a prompt for clinicians to review MRI scan results with patients, (3) greater detail about physiotherapy treatment, (4) signposting for local mental health services, (5) additional detail about the surgical procedure and potential additional procedures, for example, tenodesis, (6) adding an arm to the spider diagram to prompt patients to ask ‘will I need to stop taking my other medication’ and (7) addition of statistical data reporting patient satisfaction following surgical or non-surgical treatment. One participant suggested deleting the information about ACL self-healing as there was not enough research to support its inclusion.

These suggestions were discussed at Trial Management Group (TMG) meetings to support refinement of the intervention and its logic model (Implementation Research Logic Model, IRLM). It was agreed that the following components were added as they were deemed to add value to the intervention while ensuring it remained concise and maintained the principles and design of the original content. The following amendments were made: (1) a prompt for signposting to local mental health services was added to the information about psychological factors (option grid, ‘what else can I do?’, page 2), (2) the question ‘will I need to stop taking my medication?’ was added under the question ‘how will treatment impact my life? (patient information leaflet, spider diagram, page 8) and (3) satisfaction with treatment data was added (patient information leaflet, page 5). One participant identified a typo in the option grid which was also corrected.

The final version of the SDM intervention (patient information leaflet and option grid) is presented in online supplemental file 9, with refinements in green text.

The IRLM was also revised and is presented in online supplemental file 10, with refinements in green text. These refinements were also supported by implementation factors identified as barriers and enablers to future normalisation, described in a separate publication.

## Discussion

The results of this study confirm that the intervention is feasible to deliver and acceptable to both patients and physiotherapists. All four feasibility outcomes were achieved, with success rates ranging from 94% to 100% across the outcomes. Indicators of effectiveness suggest the intervention’s potential utility in supporting SDM processes for treatment decision-making following an ACL rupture. A full trial exploring the effectiveness of the SDM intervention is feasible.

### Feasibility and acceptability

#### Recruitment rate

The overall recruitment rate of 95.2% was achieved ahead of schedule within 4½ months. This is encouraging and suggests patients are willing to consent to participate in a trial of this nature. During qualitative interviews, patients and physiotherapists reflected on the low burden of the intervention, timing at which it was delivered and appropriateness for a wide scope of patients following ACL rupture as eligibility criteria allowed for inclusion of participants with both acute and chronic ACL ruptures aged 18 and older. These factors likely aided recruitment and so a similar strategy in a future trial is warranted. It is, however, important to highlight the high recruitment rate in the context of other musculoskeletal and orthopaedic research which is typically less than 50%.^18 19^

#### Fidelity to the intervention

Fidelity of intervention delivery was 100% for all 11 physiotherapists involved in the study. Ahead of a participants’ appointment, physiotherapists’ were sent an email reminder of key procedures, likely contributing to high fidelity. Fidelity was assessed via a self-reported checklist by the treating physiotherapist; however, this was not externally verified for accuracy. Observation of consultations in a future study would offer clarity regarding fidelity of delivery.

#### Acceptability of the intervention

Overall acceptability was 93.67% across the 15 items in the acceptability questionnaire. A recent study exploring patient acceptability of a decision aid following Achilles tendon rupture (n=13) reported similar positive responses among patients regarding acceptability.^20^ This suggests patients following a musculoskeletal/orthopaedic injury respond well to interventions supporting SDM and demonstrates appetite for future research in this area. A key prerequisite to decision-making, and essential to SDM, is to ensure that patients are aware of the treatment options available to them.^21^ In this study, 100% of patients felt ‘the presentation of information was ‘balanced’ and fair towards each treatment option’. However, the lowest scoring question at 80% was that ‘the presentation of information seemed in favour of having surgery’ which conflicts with the previous response. Interviews with patients revealed that although participants viewed the wording to be balanced, they felt research available to support a decision to proceed non-operatively was lacking. In addition, some participants described consultations which lacked clinical equipoise leaving patients uncertain about the validity of non-operative treatment. Similar to the previously discussed point, observation of consultations in a future trial may offer greater depth to the presentation of equipoise and impacts on SDM.

#### Follow-up questionnaire completion

Follow-up outcome measure completion was 100% with acceptability of online methods via text and email links. Participants received a £20 voucher of their choice on receipt of questionnaire responses which may have contributed to the high response rate. During patient interviews, participants stated that the questionnaires were not burdensome, were relevant and easy to complete following the hyperlinks sent via text or email.

### Indicators of effectiveness and qualitative exploration

Although it was not the focus of the feasibility study to determine effectiveness (nor was it powered to do so), the SWD scale was used to explore indicators of the intervention’s effect on decision-making processes. The average SWD score was 24.85 (95% CI 23.06 to 26.64, SD 3.82). Only one participant ‘disagreed’ with one statement regarding being adequately informed but did not respond to an interview invitation, limiting further exploration. Interviews with another participant, who indicated ‘neither agree nor disagree’ with the same statement suggested this was due to limited information available in the literature about the non-operative treatment pathway (as discussed above). As this study was with a small number of participants, this will be an important avenue to consider in a future larger trial to understand potential unmet needs.

Patients reflected on the intervention’s utility in providing them with access to healthcare language and the subsequent ability to ask questions deemed relevant to them. NHS England outlines current literacy levels in the UK as a crisis impacting on people’s health.^22^ In England, a 2018 survey revealed 16.4% of the population to have poor literacy skills, defined as reading at or below the level of a 9-year-old.^23^ A further 43% are thought to not understand written health information, with this rising to 60% when numbers and statistics are included.^22 24^ The inclusion of statistical data was viewed favourably by both patients and physiotherapists and there was no significant difference in outcomes for those identified to be at risk of poor health literacy. Assessing health literacy is an important element of a future trial to further understand the utility of the intervention in those with lower health literacy and levels of education.

There was some evidence of uncertainty with decision-making about treatment. One participant reported difficulty with the lack of clarity offered by the statistical data included in the intervention and discussed the challenge of making an informed decision. Uncertainty with decision-making in usual care following an ACL rupture has previously been reported, in addition to uncertainty with the non-operative treatment pathway.^6 25^ It is unknown how the participant in this feasibility study would have experienced the usual care pathway and whether the intervention had no effect on, introduced or added to existing uncertainty. Evidence from other patients suggested that the intervention did not introduce doubt, but instead offered transparency to the treatment pathway which was reflected on positively. Although previous research has identified most patients value the opportunity to be involved in decision-making, some may require more support and greater time for this process than others.^26 27^ The patient demonstrating uncertainty with decision-making in this study identified that they were likely to have responded differently (and more positively) to the follow-up questionnaires (acceptability and SWD) had they have been given more time to engage with the intervention before appraising it. Participation in decision-making has been associated with improved health outcomes and overall greater satisfaction with the consultation process.^28 29^ Understanding change in SWD scores over time and in relation to specific points of interaction may therefore be important components of a future trial.

Most participants expected the outcomes of ACLR to be better than those detailed in the intervention. This expectation has previously been reported in an Australian cohort study, where only 24% of patients had returned to their pre-injury level of sport at 1 year despite 91% expecting to when asked preoperatively.^30^ A 2023 predictors review identified patients’ positive estimation of their ability to return as a predictor of a successful return to physical activity.^31^ However, it was highlighted that clinicians should take caution to ensure patients’ expectations are not enhanced above levels that are realistic. This is an important role of the SDM intervention, with evidence from this study that engagement offered a pragmatic view of treatment outcomes that would have otherwise been assumed to be higher.

Clinician time constraints have previously been identified as a major barrier to implementing SDM.^32^ A 2016 knee osteoarthritis SDM study reported no difference in overall consultation time when using an option grid compared with usual care.^7^ One therapist with experience of a 30-minute and 60-minute consultation in the present study acknowledged that while 60 minutes was preferred, delivery was achievable within 30 minutes. Reports from both patients and physiotherapists confirmed low burden of the intervention with no perceived negative effects to consultation time. This suggests that the intervention was appropriate for use during usual physiotherapy practice. One patient participant felt SDM was not prioritised by the physiotherapist due to time constraints. Exploration in a future full trial would confirm the intervention’s effectiveness in supporting SDM processes, how it is prioritised by clinicians and its impact on consultation length.

### Strengths, limitations and reflexive considerations

Data were collected on participants’ ethnicity, recognised in the 2022 (most recent) UK National Ligament Registry (NLR) report as a future priority to support understanding of the epidemiology and outcomes following ACL rupture.^33^ While we acknowledge the present study included only 20 participants in one area of the UK, capturing this data may support future understanding of the demographics of those with ACL ruptures. Without consistent reporting of ethnic background in the literature, it is unclear whether the findings of this study are transferable to the wider UK ACL rupture population. The population included those from a wide age range, with varying levels of pre-injury and current physical activity, who sustained their ACL rupture via contact and non-contact mechanisms. These demographics would suggest that the study captured an appropriate sample encompassing a range of characteristics representative of the reported adult ACL population in the current literature.^9^

A second strength of this study is that participants’ level of deprivation, level of education and health literacy were recorded, to support understanding of the SDM interventions utility in those at risk of poorer healthcare experiences and outcomes. While no significant differences were detected between groups, this only offers an indication of the intervention’s utility in SDM process as the study was not powered to detect effectiveness. This is an important area of exploration in a future trial to ensure individuals from lower socioeconomic backgrounds are not at risk of marginalisation from SDM processes. All participants identified to be at risk of poor health literacy declined to be contacted to share their experiences in a qualitative interview. We therefore may not have captured a full range of perceptions and experiences, particularly of those at risk of poor health literacy.

Although there were provisions in place to recruit non-English speaking participants, none were identified as eligible for the trial. Future consideration to understand how the material may translate into other languages and its utility with non-English speaking participants may therefore be needed.

Further, the qualitative interviews were carried out by the lead researcher who also identifies as a clinician at one of the physiotherapy departments running the study and who has previously practiced at the second department. The lead researcher has been a member of the clinical team for several years. This may have limited the discussions with physiotherapists who may not have felt comfortable accounting negative experiences to a clinical colleague.

## Conclusion

All four feasibility outcomes were achieved, with success rates ranging from 94% to 100% across the outcomes. This study has demonstrated that implementation of an SDM intervention in the ACL rupture pathway is acceptable to both patients and physiotherapists and can be delivered with high fidelity. Exploring indicators of effectiveness, through quantitative and qualitative data, identified the interventions’ utility in improving patient knowledge, supporting patients to understand medical terminology to formulate clinically relevant questions, facilitating SDM conversations, empowering patients to be involved in decision-making about their treatment and providing up-to-date clinically relevant information for both patients and clinicians. These findings support the need for further evaluation in a definitive trial, given the importance of SDM in ensuring patient-centred care in ACL injury management, as evidence to date highlights that surgical intervention is not necessary for all.

## Supplementary material

10.1136/bmjopen-2024-095189 online supplemental file 1

10.1136/bmjopen-2024-095189 online supplemental file 2

10.1136/bmjopen-2024-095189 online supplemental file 3

10.1136/bmjopen-2024-095189 online supplemental file 4

10.1136/bmjopen-2024-095189 online supplemental file 5

10.1136/bmjopen-2024-095189 online supplemental file 6

10.1136/bmjopen-2024-095189 online supplemental file 7

10.1136/bmjopen-2024-095189 online supplemental file 8

10.1136/bmjopen-2024-095189 online supplemental file 9

10.1136/bmjopen-2024-095189 online supplemental file 10

## Data Availability

All data relevant to the study are included in the article or uploaded as supplementary information.
