# Supplementary material for: Use of a shared decision-making intervention to support treatment decision-making for patients following an anterior cruciate ligament rupture: a mixed methods feasibility study
Source: BMJ Open. 2025 Aug 27;15(8):e095189. doi: 10.1136/bmjopen-2024-095189 (PMC12406910; doi:10.1136/bmjopen-2024-095189)
Supplement: online supplemental file 4 [file bmjopen-15-8-s004.pdf]

The POP-ACLR Study

Acceptability Questionnaire

Please tick whether you **agree or disagree** with the following statements. Think about each statement in relation to the **shared decision making tools** you have used to help you decide on the **treatment of your ACL injury**.

	Agree	Disagree
The information in the shared decision making tools was **helpful**		
The information helped me to **understand my ACL tear**		
The information helped me to **understand my treatment options**		
The diagram on page 8 of the information leaflet was helpful in getting me to **think about key questions** to discuss with my healthcare professional		
The **option grid** helped me to **make a decision**		
I am aware of the **advantages** of each **treatment option** for my ACL injury		
I am aware of the **disadvantages** of each **treatment option** for my ACL injury		
I **understood** all the information in the **shared decision making tools**		
The **length** of the shared decision making tools was '**just right**'		
The **length** of the shared decision making tools was '**too short**'		
The **length** of the shared decision making tools was '**too long**'		
The presentation of information seemed in **favour of having surgery**		
The presentation of information seemed in **favour of not having surgery**		
The presentation of information was '**balanced'** and **fair** towards each treatment option		
I **would recommend** the shared decision making tools to **other patients** in a similar position to me		
