# Supplementary material for: Use of a shared decision-making intervention to support treatment decision-making for patients following an anterior cruciate ligament rupture: a mixed methods feasibility study
Source: BMJ Open. 2025 Aug 27;15(8):e095189. doi: 10.1136/bmjopen-2024-095189 (PMC12406910; doi:10.1136/bmjopen-2024-095189)
Supplement: online supplemental file 5 [file bmjopen-15-8-s005.pdf]

Satisfaction with Decision (SWD) Scale for ACL injury treatment decision

Please answer the following questions about your decision about treatment for you ACL injury. Please indicate to what extend each statement is true for you **AT THIS TIME** (circle one number for each statement).

	Strongly disagree	Disagree	Neither agree nor disagree	Agree	Strongly agree
I was adequately informed about the different treatments available for my ACL injury	1	2	3	4	5
The decision I made was the best decision possible for me personally	1	2	3	4	5
My decision was consistent with my personal values	1	2	3	4	5
I expect to successfully carry out(or continue to carry out) the decision I made	1	2	3	4	5
I had as much input as I wanted in the choice of treatment for my ACL injury	1	2	3	4	5
I am satisfied with the decision that was made about treatment for my ACL injury	1	2	3	4	5
