# Supplementary material for: Use of a shared decision-making intervention to support treatment decision-making for patients following an anterior cruciate ligament rupture: a mixed methods feasibility study
Source: BMJ Open. 2025 Aug 27;15(8):e095189. doi: 10.1136/bmjopen-2024-095189 (PMC12406910; doi:10.1136/bmjopen-2024-095189)
Supplement: online supplemental file 6 [file bmjopen-15-8-s006.docx]

**Interview Topic Guide – Participant / Clinician Interview**(delete as appropriate for interviewee)
Version 1.0 – 23/10/2023

# **Introduction and Background**

- Thank you for agreeing to take part in the study to discuss use of the shared decision making tools
- Tell me about your experience of using the tools

## Possible prompts:

- Helpfulness of tools
- Length / readability / layout

# **Topic 1: Acceptability**

## Possible prompts:

- Acceptability of using the tools
- Thoughts on acceptability of use in practice
- Where they helpful in decision making about treatment
- Format – paper vs online

# **Topic 2: Implementation Factors**

## Possible prompts:

- Where the tools burdensome to use?
- For participants only
  - Where you happy using the tools with the physiotherapist? Whose role do you think it should be?
  - Did you feel the tools allowed for a consultation and discussion specific to you and your goals
- For clinicians only
  - Was the education you received in training sufficient to use the tools?
  - How you do envision the tools being used in practice?
  - Were the tools compatible with current practice?
  - Did you feel able to use the tools with every participants e.g were you able to adapt/tailor consultations using them
  - Did you feel like it was your role to deliver the tools?
  - How do you think we should be evaluating the tools?

# **[Clinicians only] Topic 3: Contamination**

## Possible prompts:

- Having used the tools – how do you think it’s impacted your practice in consultations where you have not used the tools (for ACL patients and other MSK conditions)

# **Close**

- Is there anything you feel could be changed about the SDM tools
- Anything further you would like to mention/discuss
- Thank you for taking the time to discuss your experience
