# Supplementary material for: Use of a shared decision-making intervention to support treatment decision-making for patients following an anterior cruciate ligament rupture: a mixed methods feasibility study
Source: BMJ Open. 2025 Aug 27;15(8):e095189. doi: 10.1136/bmjopen-2024-095189 (PMC12406910; doi:10.1136/bmjopen-2024-095189)
Supplement: online supplemental file 7 [file bmjopen-15-8-s007.docx]

Interview Participant Demographics

| **Patient Participants** | |
| --- | --- |
| Age, median | 32 |
| Sex, n  Female  Male | 2  3 |
| Ethnicity, n  White British  Asian Pakistani | 4  1 |
| Level of education, range | 2-4 |
| REALM-R, range | 7-8 |
| ACL injury mechanism, n  Contact  Non-contact | 2  3 |
| Time between injury and diagnosis, range | 5 days to 6 years |
| Pre-injury physical activity level, n  Competitive – national level  Competitive – regional level  Recreational/Leisure | 1  2  2 |
| Pre-injury physical activity frequency, n  2-4 times a week  5-7 times a week | 2  3 |
| Employment status, n  Full time  Part-time  Student | 3  1  1 |

Table 1 - Patient participants

| **Physiotherapy Participants** | |
| --- | --- |
| Agenda for change grade, n  Band 5  Band 6  Band 7 | 1  3  1 |
| Treating site, n  FNCH  QHB | 3  2 |
| Number of patients treated in the trial, n  1  2  3 | 2  1  2 |
| Consultation length, n  30 minutes  60 minutes  30 and 60 minutes | 3  1  1 |
| Consultation medium, n  Face-to-face  Telephone call | 3  1 |

Table 2 - Physiotherapist participants

* Band 5 – newly qualified/junior, Band 6 – senior, Band 7 – specialist
