# Supplementary material for: Use of a shared decision-making intervention to support treatment decision-making for patients following an anterior cruciate ligament rupture: a mixed methods feasibility study
Source: BMJ Open. 2025 Aug 27;15(8):e095189. doi: 10.1136/bmjopen-2024-095189 (PMC12406910; doi:10.1136/bmjopen-2024-095189)
Supplement: online supplemental file 8 [file bmjopen-15-8-s008.docx]

Nested Qualitative Interviews – Supporting Quotes

Quote 1

“It’s very precise. There’s not masses of information that aren’t relevant, but then I also think it tailors to everyone” [P0012, patient]

Quote 2

“the main thing that stands out in my mind is the chance of re-rupture … You know, it’s quite a scary statistic, and also the stats about people getting back to playing sport. I mean, quite worrying. But I understand that, you know, those are figures and you can't lie about them, and to be honest I’m grateful to have seen it early on because there’s no smoke and mirrors” [P0012, patient]

Quote 3

“my physio was like really good … I went away from the first time seeing him like I opened the book of knowledge … anything I asked him, he just explained in depth and made me understand” [P0011, patient]

Quote 4

“If I wasn’t given this leaflet, I think I would be quite clueless at to what’s happening… I feel like I’m in a position where I can make a decision” [P0015, patient]

Quote 5

“If it'd been more conclusive like, oh yes, it’s a hundred percent success rate, that would've been easier … if the outcomes had been more compelling, but they are what they are.” [P0009, patient]

Quote 6

“It’s got some really good statistics in as well that are helping me see. It’s not a one-size-fits-all approach.” [P0015, patient]

Quote 7

“these are a lot of the questions that patients ask you. We should have been able to answer before, but this gave clarity and data to those conversations, which was useful for patients.” [P0022, physiotherapist]

Quote 8

“I haven’t Googled it, no. …I don’t think I even feel the need to go and watch, or research anything, because I just believe what I see in the booklet.” [P0011, patient]

Quote 9

“patients arrived more informed, which therefore streamlined the appointments and the discussions … It helped me as a clinician with quick and structured and accessible advice, based on their questions. Rather than rambling on you could just summarise nicely and when you’ve not seen an ACL rupture for a while it was useful to have the reference point, for particularly the research side of things.” [0022, physiotherapist]

Quote 10

“As a non-medical person, it gives you an understanding so you can speak to medical people about these things … because I have no clue basically. And then once you … learn a little bit about it you can ask questions about what’s going on … it has given me a better foundation to have a conversation … instead of just saying is the knee okay?” [P0011, patient]

Quote 11

“taking part in this study has … encouraged [shared decision-making conversations] a little bit more … having more of an open dialogue in terms of actually what that patient wishes. What are the options available to that patient and what is it that they’re thinking at that time.” [P0023, physiotherapist]
