# Supplementary material for: Use of a shared decision-making intervention to support treatment decision-making for patients following an anterior cruciate ligament rupture: a mixed methods feasibility study
Source: BMJ Open. 2025 Aug 27;15(8):e095189. doi: 10.1136/bmjopen-2024-095189 (PMC12406910; doi:10.1136/bmjopen-2024-095189)
Supplement: online supplemental file 9 [file bmjopen-15-8-s009.pdf]

Anterior Cruciate Ligament (ACL) Rupture and Treatment

Patient Information Leaflet

POP-ACLR

The POP-ACLR Study

Authors: Hayley Carter, David Beard, Paul Leighton, Fiona Moffatt, Benjamin Smith, Kate Webster, POP-ACLR consensus group*, Pip Logan.

***Consensus group**: Scott Backler, George Crouchley, Rebecca Cussens, Charlotte Dodsley, Matthew Gane, Sarah Holt, Josh McCallion, Thomas Kurien, Justin Murr and Michelle Slack.

Acknowledgements: This leaflet was produced as part of the POP-ACLR research consensus study. The consensus team contributed to the overall content to be included and we acknowledge their valuable input.

Funding: Hayley Carter, Clinical Doctoral Research Fellow, NIHR302104 is funded by Health Education England (HEE) / NIHR for this research project. The views expressed in this leaflet are those of the author(s) and not necessarily those of the NIHR, NHS or the UK Department of Health and Social Care.

FUNDED BY NIHR | National Institute for Health and Care Research

NHS University Hospitals of Derby and Burton NHS Foundation Trust

University of Nottingham UK | CHINA | MALAYSIA

Table of Contents

Introduction 2

What is the ACL? 2

What are the treatment options? 3

1. Surgery *3*

2. Non-surgery *3*

Other options *4*

What are the outcomes of each treatment option? 4

Return to preinjury activity level *4*

Return to sport *4*

Return to work *5*

Return to driving *5*

Other *5*

What are the risks? 6

What to expect 7

What to think about 7

What next? 8

Other information 8

References 9

Notes 10

Introduction

This information leaflet is designed to provide you with essential information about your Anterior Cruciate Ligament (ACL) injury and your treatment options.

This leaflet should be used alongside the **Option Grid**.

What is the ACL?

The ACL is one of two ligaments that cross inside the knee joint. It attaches between your thigh bone (femur) and shin bone (tibia). The ACL's main job is to provide stability to the knee joint. **Figure 1**, shows an ACL in the left knee.

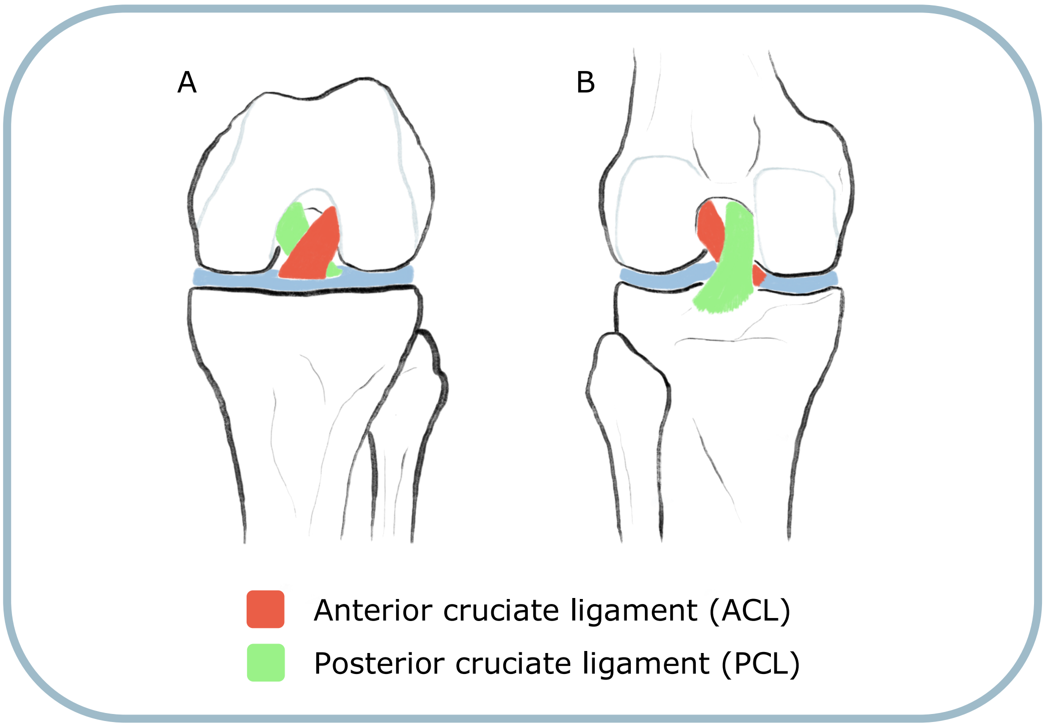

Figure 1 - Left knee joint

A. Front view of the left knee with the knee bent to 90 degrees
B. Back view of the left knee joint with the knee fully straight

ACL ruptures (also referred to as tears) are common. They can cause pain, swelling and difficulty with knee function. A rupture can also cause instability of the knee, which can be more of a problem for some people than others.

What are the treatment options?

In the UK, there are two main treatment options:

1. **Surgery**
2. **Non-surgery** (also referred to as rehabilitation)

Three research trials have compared ACL reconstruction surgery (ACLR) with rehabilitation (non-surgery). Only one of these was in a UK, NHS setting.

The oldest trial, from 2010, found no difference between surgery and rehabilitation.[1] The researchers suggested that surgery may be unnecessary for some. They recommended that rehabilitation should be trialled first. The second trial, from 2021, compared early ACLR to rehabilitation with delayed ACLR. The researchers of this trial reported early surgery to be better.[2] The final trial, completed in the UK in 2022, reported outcomes for those having surgery to be better.[3]

These results show that there isn't a one-size fits all approach when it comes to ACL injury management. This is why making a decision that is right for you is important. Patients involved in developing this leaflet felt it was important to know that ACL treatment (surgery and non-surgery) is both physically and mentally challenging. Both treatment options require time and effort. It is important you are aware what both treatments involve. The following information provides some more detail about each option.

1. Surgery

Surgery involves **reconstructing the torn ligament with a graft.** This graft is usually taken from another part of your body. The graft most used in the UK is a piece of tendon (part of the muscle) from your hamstring. However, a graft can also be taken from the tendon in your knee or from a donor.

If you decide to have surgery, you will have a conversation with your surgeon about which graft is best for you.

After your ACL reconstruction you will need to undergo a progressive rehabilitation programme with a physiotherapist. This will take at least 6-months and depending on your goal (e.g. if you wish to return to competitive sports), may take longer than 12-months.

2. Non-surgery

Non-surgery involves **rehabilitation with a therapist**, such as a physiotherapist or sports rehabilitator. This will involve exercises that aim to improve your walking, knee range of movement, strength and stability. Although there is some similarity with everyone's rehabilitation, your rehabilitation will be specific to you and depend upon your symptoms and ability.

Other options

There has been some early evidence that the ACL may be able to heal itself, without surgery. This research, from 2022, showed that 32 patients had evidence of some ACL healing on an MRI scan after 2 years.[4] At this stage we cannot say that your ACL would heal itself without surgery. The evidence is not yet strong enough.

What are the outcomes of each treatment option?

Most patients want to know when they can return to work, physical activity, sport and driving. **Table 1** shows some statistics taken from research studies to answer these questions.

Further information is discussed below the table.

	**Surgery**	**Rehabilitation**
Return to preinjury activity level	28% by 18-months	24% by 18-months
Return to sport	55% returned by 40 months	No research available
Work	2.5 months (average, job dependent)	No research available but job dependent
Driving	Typically 6-weeks	No set restriction
Satisfaction	83% felt their knee was better than before treatment	68% felt their knee was better than before treatment

Table 1 - Treatment outcomes

Return to preinjury activity level

Returning to your preinjury activity level means going back to your activities 'as normal'. For example, returning to the same level football league as you were playing in before your injury. The percentages in the table are from a 2022 UK trial.[3] Other research trials have shown that for those having surgery:

- **24%** have returned to their preinjury levels of activity **after 1-year**
- Less than **45%** have returned at **2-years**.[5,6]

Return to sport

Return to sport means that you have gone back to your sport but may not be competing at the same level.

There is no research reporting the number of people who return to sport after rehabilitation. A 2014 study (that also included people having revision ACL

surgery) reported **55%** returned to competitive sport **40 months after surgery**.[7]

Return to work

Return to work hasn't been reported as frequently in the research as return to sport. It also varies depending on how physically active/labour intense your job is. A 2017 study completed in the Netherlands reported that on average, patients had returned to work by **78 days after surgery**.[8] Returning after 78 days was most likely for those in more labour-intensive jobs or those who needed crutches for longer after surgery.

There is no data to suggest when you can return to work if you choose not to have surgery. If you're currently off work and choose not to have surgery, your clinician (e.g. physiotherapist or occupational therapist) will support you in returning when it is right for you.

Return to driving

There is no data to suggest when people return to driving after surgery or rehabilitation.

Typically, you can return to driving 6-weeks after your operation. Although there are no set limitations, you can return when you feel safe and are able to carry out all emergency procedures. Your car insurance company may have set limitations for when you can return, so it is worth checking with them too.

If you choose not to have surgery, your clinician will support you to return to driving.

Satisfaction

In the 2022 UK trial comparing ACL surgery with rehabilitation, patients were asked about satisfaction of their treatment at an 18-month follow-up.

Surgery
Of those who were treated with surgery:
- 83% said their knee was better than before treatment.
- 80% said they would choose surgery again, 5% said they would not.

Rehabilitation
Of those who were treated with rehabilitation:
- 68% said their knee was better than before treatment.
- 61% said they would choose rehabilitation again, 18% said they would not.

Other

As the research around ACL self-healing is new, there are no statistics to draw upon in the research to answer any of the above questions.

What are the risks?

There are some risks associated with having surgery which include:

Re-rupture

There is a chance the graft could **re-rupture** after surgery. A 2022 study reported 7.2% of patients underwent revision surgery within 9 years of their first surgery due to a re-rupture.[9]

Complications of surgery

Some patients may develop a '**cyclops lesion**' (a ball of excess graft tissue at the front of the ACL graft). The chance of this occurring is approximately 21-35% and is more common in women.[10] Cyclops lesions can require further surgery to be removed. Once removed, patients typically make a full recovery.

There are also risks associated with having surgery such as **infection** (less than 2%) and a **blood clot** (less than 1%).[11]

Donor site pain/discomfort

You may experience some **pain/discomfort** where your donor graft has been taken from (e.g. hamstring).

Osteoarthritis and further injury

It is a common thought that surgery protects against the development of **osteoarthritis**. However, a 2022 study found no difference in the rate of osteoarthritis between those who did or did not have surgery.[12] Whether you go on to have surgery or not, you are at an increased risk of developing osteoarthritis because of your ACL injury.

It is also thought that without surgery, you are at risk of a **meniscal (cartilage) injury**. The evidence about this is unclear. We cannot say for sure that surgery prevents further meniscal injury.[13]

Instability

If you choose not to have surgery, there is a chance you **may not regain stability** in your knee. This might mean you are unable to return to the activities you want to. Where this is the case, you will need to discuss the option of surgery with your clinician. In research studies comparing rehabilitation with surgery, approximately 50% of patients who had rehabilitation first, went on to have surgery later.[1–3]

Your clinician will discuss these risks with you.

What to expect

The typical patient journey for surgery and rehabilitation is outlined in **Figure 2**.

Surgical Pathway

1. Listed for surgery
2. Outpatient physiotherapy
3. Preoperation appointment
4. Contacted for a date for your surgery
5. Surgery
6. Outpatient physiotherapy
7. Post-operation orthopaedic appointment
8. Discharge from orthopaedics (open appointment)
9. Discharge from physiotherapy (open appointment)

Rehabilitation Pathway

1. Referred to physiotherapy
2. Outpatient physiotherapy
3. Ongoing physiotherapy
4. Discharge from physiotherapy (open appointment)

Figure 2 - Expected journey

We recommend discussing expected timeframes for each step with your clinician.

What to think about

The ‘what should I be thinking about’ diagram (next page) outlines some key questions to think about. You should discuss these with your clinician. These will help you to make a decision about what treatment is best for you.

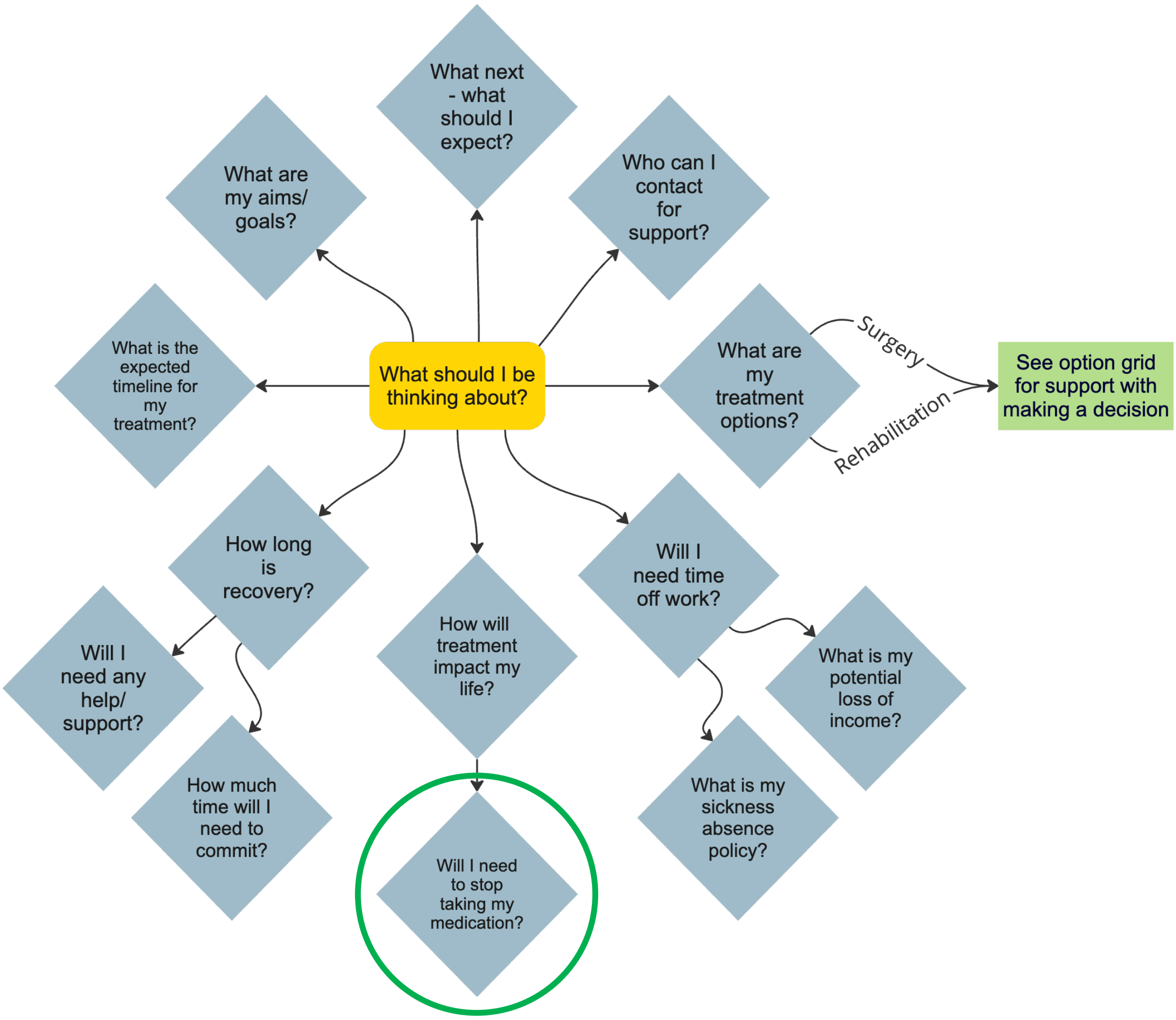

What next?

You will need to make a decision about what treatment is best for you. At present, the evidence isn't strong enough for us to conclude that surgery is necessary for everyone or that rehabilitation is the best option.

This information leaflet aims to provide you with the necessary information to help you understand your options.

The **Option Grid** should be used with your clinician(s) to help you decide on the treatment that is right for you.

Other information

There is a lot of information about ACL injuries and treatment available online. Some of this information is more accurate than others. If you read anything online that doesn't fit with what your healthcare professional is telling you or that you have read here, you should ask them about it.

References

Below are links to the research studies mentioned in this leaflet. The number next to the sentence in the leaflet represents the research the finding was taken from.

1 Frobell RB, Roos EM, Roos HP, Ranstam J, Lohmander LS. A Randomized Trial of Treatment for Acute Anterior Cruciate Ligament Tears. *New England Journal of Medicine* 2010; **363**: 331–42.

2 Reijman M, Eggerding V, Van Es E, *et al.* Early surgical reconstruction versus rehabilitation with elective delayed reconstruction for patients with anterior cruciate ligament rupture: COMPARE randomised controlled trial. *The BMJ* 2021; **372**. DOI:10.1136/bmj.n375.

3 Beard DJ, Davies L, Cook JA, *et al.* Rehabilitation versus surgical reconstruction for non-acute anterior cruciate ligament injury (ACL SNNAP): a pragmatic randomised controlled trial. *The Lancet* 2022; **400**: 605–15.

4 Filbay SRose, Roemer FW, Lohmander LStefan, *et al.* Evidence of ACL healing on MRI following ACL rupture treated with rehabilitation alone may be associated with better patient-reported outcomes: a secondary analysis from the KANON trial. *Br J Sports Med* 2022; **0**: 1–9.

5 Dunn WR, Spindler KP. Predictors of activity level 2 years after anterior cruciate ligament reconstruction (ACLR): A multicenter orthopaedic outcomes network (MOON) ACLR cohort study. *American Journal of Sports Medicine* 2010; **38**: 2040–50.

6 Ardern C, Taylor N, Feller J, Whitehead T, Webster K. Sports participation 2 years after anterior cruciate ligament reconstruction in athletes who had not returned to sport at 1 year: A prospective follow-up of physical function and psychological factors in 122 athletes. *American Journal of Sports Medicine* 2015; **43**: 848–56.

7 Ardern C, Taylor N, Feller J, Webster K. Fifty-five per cent return to competitive sport following anterior cruciate ligament reconstruction surgery: An updated systematic review and meta-analysis including aspects of physical functioning and contextual factors. *Br J Sports Med* 2014; **48**: 1543–52.

8 Groot JAM, Jonkers FJ, Kievit AJ, Kuijer PPFM, Hoozemans MJM. Beneficial and limiting factors for return to work following anterior cruciate ligament reconstruction: a retrospective cohort study. *Arch Orthop Trauma Surg* 2017; **137**: 155.

9 Randsborg P-H, Cepeda N, Adamec D, Rodeo SA, Ranawat A, Pearle AD. Patient-Reported Outcome, Return to Sport, and Revision Rates 7-9 Years After Anterior Cruciate Ligament Reconstruction Results From a Cohort of 2042 Patients. 2022. DOI:10.1177/03635465211060333.

10 Kambhampati SBS, Gollamudi S, Shanmugasundaram S, Josyula VVS. Cyclops Lesions of the Knee: A Narrative Review of the Literature. *Orthop J Sports Med* 2020; **8**: 2325967120945671.

11 Ding DY, Tucker LY, Rugg CM. Comparison of Anterior Cruciate Ligament Tears Treated Nonoperatively Versus With Reconstruction: Risk of Subsequent Surgery. *American Journal of Sports Medicine* 2022; **50**: 652–61.

12 Webster KE, Hewett TE. Anterior Cruciate Ligament Injury and Knee Osteoarthritis: An Umbrella Systematic Review and Meta-analysis. Clinical Journal of Sport Medicine. 2022; **32**: 145–52.

13 Filbay SR. Early ACL reconstruction is required to prevent additional knee injury: a misconception not supported by high-quality evidence. *Br J Sports Med* 2019; **53**. DOI:10.1136/bjsports-2018-099842.

Notes

....

....

....

....

....

....

....

....

....

....

....

Making a Decision about Anterior Cruciate Ligament Injury Treatment

This option grid presents evidence-based information about treatment for an anterior cruciate ligament (ACL) rupture. You should use this with your healthcare professional to help you make the right decision for you.

	**Surgery**<br>**(ACL reconstruction)**	**Rehabilitation**<br>**(non-operative management)**
**What are my options?**	Surgery involves reconstructing your torn ACL with a graft taken from another part of your body (typically a tendon from your hamstring or knee). Sometimes a donor graft may be used.	Rehabilitation involves working with a therapist. The areas targeted during treatment are specific to you and your injury but typically aim to improve your overall fitness, knee function, strength and stability.
**What do I need to know about this treatment?**	Surgery involves a keyhole operation to reconstruct your torn ACL with a graft. This takes approximately 1 hour and will require you to be under local or general anesthetic.<br><br>You may need to wear a brace after surgery and use elbow crutches.	Rehabilitation requires you to commit time to a regular exercise programme. This may be in a one-to-one or group setting. Your therapy sessions are unlikely to be supervised for the duration of your treatment and you will need to complete your exercises independently at home or in a gym.
**What does the research say about this treatment option?**	A trial completed in the UK (2022) reported surgery to be better than rehabilitation for patients with an ACL tear older than 4-months. In this trial, it was concluded that those who had surgery had better scores 18-months after surgery for knee outcomes, quality of life and activity level.<br><br>However, 2 previous trials have concluded that:<br>▪ surgery may be unnecessary for some and rehabilitation should be trialed first<br>▪ early reconstruction may be better than delayed reconstruction<br><br>Both trials were completed outside the UK in different healthcare settings.	The UK 2022 trial reported that 41% of patients went on to have surgery after a period of rehabilitation.<br><br>There has previously been no consensus on the best rehabilitation to offer to patients. Because of this, treatment has varied across the UK and in research studies. In 2023 a group of patients and clinicians agreed some recommendations for rehabilitation to help improve and standardise treatment.<br><br>We know that some patients manage well without surgery. At present (2023) it is unclear who is an ideal candidate for surgery and who may manage without it.
**What are the outcomes of this treatment?**	**Preinjury activity level** - 20% of patients return to their preinjury levels of activity 1-year after surgery, 28% return at 18-months and less than 45% return at 2-years.<br><br>**Work** – The average time to return to work after ACL reconstruction is approximately 2 and a half months. This may be shorter for desk-based jobs and longer for more labour-intensive roles.<br><br>**Driving** – Typically you can return to driving 6-weeks after your operation.	**Preinjury activity level** - 24% of patients return to their preinjury activity level 18-months after rehabilitation.<br><br>**Work** – no research to date has collected information on the time to return to work following ACL injury for patients who chose non-operative treatment.<br><br>**Driving** – there is no set restriction on returning to driving after an ACL rupture. This should be decided by you and your clinician.
**If I choose this treatment, what happens next?**	If you decide to have surgery, you will be placed on the waiting list. Your clinician should be able to give you an estimated waiting time. Your surgeon will also discuss your graft choice with you. You should also be referred to physiotherapy for support whilst you wait for surgery.	You will be referred to physiotherapy, your clinician should be able to give you an estimated waiting time.<br><br>You will work with your physiotherapist to develop some goals to work towards. You should start thinking about these now, ready to discuss at your first appointment.

**What are the risks?**	The risks associated with surgery include:<br>▪ Re-rupture (7.2%)<br>▪ Cyclops lesion (21-35%)<br>▪ Infection (less than 2%)<br>▪ Blood clot (less than 1%)<br>▪ Donor site pain/discomfort<br>▪ Numbness around the knee<br><br>There is no increased risk of osteoarthritis should you decide to have surgery.	The risks associated with rehabilitation include:<br>▪ Ongoing, symptomatic instability<br>▪ Potential for further injury (unclear risk)<br><br>It is a common thought that, without surgery, you are at risk of a meniscal (cartilage) injury. There is evidence to suggest this may or may not be true. As of 2023, the evidence is unclear.<br><br>There is no increased risk of osteoarthritis should you decide not to have surgery.
**Will I need to have more treatment?**	After your ACL reconstruction you will need to undergo a progressive rehabilitation programme with a physiotherapist. This will take at least 6-months and depending on your goal (e.g. if you wish to return to competitive sports), may take longer than 12-months.<br><br>If complications arise, further surgery may be needed. Otherwise, the graft remains in place and does not need to be replaced in the future unless you have a second injury.	If you do not return to your desired functional level, you can discuss the option of surgery with your clinician.
**Are there any other options?**	**Repair**<br>There is limited evidence that ACL repair is a successful surgical option to treat an ACL rupture. ACL repair is a technique that does not use a graft and instead repairs the torn ligament with stitches.<br><br>Research in this area is limited and so at present (2023), we do not have enough evidence to suggest the success of this treatment.	**Self-healing**<br>New evidence was published in 2022 showing the ACL to have healed (to some extent) without surgery. This has been reported in a small number of patients and the long-term effects are mostly unknown.<br><br>Research in this area is limited and so at present (2023), we do not have enough evidence to suggest the success of this treatment.
**What else can I do?**	It is important that you consider the following:<br>▪ **Fitness management** – your clinician may discuss your overall health and fitness with you. This may include your cardiovascular health and weight management to support you in the recovery of your ACL rupture and/or help you prepare for surgery.<br><br>▪ **Psychological factors** – research suggests that psychological barriers, such as fear of reinjury, are the most common reason for patients failing to return to their preinjury level of activity. There is also research to suggest that some psychological factors, measured before surgery, can predict your return to preinjury levels after surgery. If you are concerned about your mental wellbeing and feel you would like further support, talk to your clinician. They can signpost you to local services and support you to access further help if needed.<br><br>▪ Optimising your **sleep and diet/nutrition** may also be important to help your recovery, if you would like further support, talk to your clinician.<br><br>▪ Take time to think about what your **end goal** is, this will help you to think about whether surgery or rehabilitation is the best option for you.	
**What does my clinician recommend?**	You may wish to gain a recommendation from more than one clinician.<br>(e.g. surgeon 1, physiotherapist, surgeon 2)	
**What is my treatment preference?**	It is important to think about what treatment you think you would like to have. Write this here to discuss with your clinician.	
