# Supplementary material for: Use of a shared decision-making intervention to support treatment decision-making for patients following an anterior cruciate ligament rupture: a mixed methods feasibility study
Source: BMJ Open. 2025 Aug 27;15(8):e095189. doi: 10.1136/bmjopen-2024-095189 (PMC12406910; doi:10.1136/bmjopen-2024-095189)
Supplement: online supplemental file 10 [file bmjopen-15-8-s010.docx]

Revised implementation research logic model (IRLM)
