# Supplementary material for: Use of a shared decision-making intervention to support treatment decision-making for patients following an anterior cruciate ligament rupture: a mixed methods feasibility study
Source: BMJ Open. 2025 Aug 27;15(8):e095189. doi: 10.1136/bmjopen-2024-095189 (PMC12406910; doi:10.1136/bmjopen-2024-095189)
Supplement: Supplementary file 11 [file bmjopen-2024-095189.reviewer_comments.pdf]

PEER REVIEW HISTORY

BMJ Open publishes all reviews undertaken for accepted manuscripts. Reviewers are asked to complete a checklist review form and are provided with free text boxes to elaborate on their assessment. These free text comments are reproduced below.

ARTICLE DETAILS

Title (Provisional)

Use of a shared decision-making intervention to support treatment decision-making for patients following an anterior cruciate ligament rupture: a mixed methods feasibility study

Authors

Carter, Hayley; Beard, David; Dodsley, Charlotte; Leighton, Paul; McCallion, Joshua; Moffatt, Fiona; Smith, Benjamin Edward; Webster, Kate E.; Logan, Phillipa

---

VERSION 1 - REVIEW

**Reviewer**	**1**
**Name**	**van Veenendaal , Haske**
**Affiliation**	**Erasmus University Rotterdam, ESHPM**
**Date**	**13-Jan-2025**
**COI**	**None**

A useful study. And exploratory. My main feedback is that I wonder whether the results are presented with too much conviction? For example, is it realistic to say that the intervention was effective in conversations between patients and clinicians? And that the intervention would have been successfully implemented? If so, how was this established?

Introduction:

- Is the last sentence the purpose of the study (This new SDM intervention..... individual qualitative conversations). Perhaps be explicit then?

Methods:

- What were the criteria for purposeful sampling?

- Physiotherapists were also trained. Why was this not mentioned as part of the intervention, since training is different in nature from applying a SDM tool? And was it training or rather an explanation with instruction?

- Can the list of PROs be included in the appendix?

- Why were existing instruments not used for the acceptance questionnaire?

- Under Sample size and Recruitment, it states that 6 plus 6 interviews will be conducted. Under Nested Qualitative Interviews, the targets are reduced to 5 plus 5?

- Data collection: Data were mapped on a matrix. Was this conducted by one researcher? Please state the number of researchers and name.

Results:

- See my earlier comment: I find the paragraph 'effectiveness' confusing. It suggests that a quantitative effect has been measured and demonstrated.

- I also find this paragraph's wording too much of an interpretation instead of the actual results of the interviews. It is stated several times that 'the SDM intervention was perceived to be effective ....'). This fits better under discussion, where then the limitations to this interpretation can be discussed. If the 1st sentence of each paragraph is omitted, the text gains clarity as far as I am concerned.

Discussion:

- The discussion is too long. This does not properly convey the core message. Based on the study aim, I would select some key findings for the discussion and mirror them on previous research and what the next steps could be.

- The results are encouraging and legitimate further research and implementation. I would better balance that positive side with modesty.

- To make it more to-the-point, I would discuss all the limitations of the research only under this paragraph and not in the text beyond it.

- This could also be better reflected in the conclusion: what can practitioners do with these results and, what steps are still needed to really establish applicability and added value for patients and practice?

**Reviewer**	**2**
**Name**	**Gamble, Andrew**
**Affiliation**	**The University of Sydney**
**Date**	**21-Apr-2025**
**COI**	**None**

I thank the authors for the opportunity to review this paper. Below are my comments:

Abstract:

If word count allows, is it possible to consider adding the following information?

Design: could this be more specific?

Participants: How was ACL rupture determined? MRI? Were they acute ACL rupture?

Outcomes: How was acceptability measured? Was this via nested qualitative interviews?

Results: How many participants were contacted? Did you include consent rate? How many were interviewed?

Introduction:

8 – It may be helpful to clarify if you are referring to acute ACL rupture when referring to the RCT conducted as this can change the interpretation of surgical vs non-surgical outcomes. You could say that non-surgical management is a viable option for acute ACL rupture based on two randomised controlled trials to show the need for a shared decision-making tool.

8- It would be better to use the term 'rupture' or 'injury' throughout

12 - How many participants were in the 'UK qualitative study'?

30 – The beginning of this paragraph focuses on the development process but does not provide the reader with enough information to understand what the shared decision-making tool being evaluated involves.

Method:

16 – This section is hard to follow. Can it be restructured to clearly state the aims of this paper compared to those described elsewhere or that will be addressed in the future?

29 – Is this a single centre study? You say across three sites here?

34 – In the abstract you use the term clinician and here physiotherapist – are all interviews with physiotherapists?

36 – What are the eligibility criteria? Is this adults only? Was it within a certain timeframe from ACL rupture? Maybe add this in a box? Or report this before saying they were invited to participate? And how were they invited? What was the process? Who contacted patients at what stage? How? Etc.

Data collection:

3 – what were the questionnaires? Is this referring to baseline only?

Intervention

17 – Could you include what evidence the SDM tool was based on?

21 – What are the questions about?

27 – A link was provide? Were they given a physical copy?

28 – What does 'digital intervention' refer to? Is this viewing the SDM tool? Or questions mentioned previously? This isn't clear.

35 – There needs to be more information about the physiotherapists involved in the trial earlier or more consistency with terminology used. Was it an objective to evaluate the training of physiotherapists involved in the trial too? There are too many terms used to describe the 'patient information leaflet' digital intervention' etc.

Feasibility

9 - This section is clearer but needs to match the information and terminology used prior.

20 – was acceptability questionnaires given after viewing the SDM tool or after consultation with the physiotherapist?

42 This could be reworded to avoid confusion that this was feedback about the SDM tool.

Nested qualitative interviews:

- The number interviewed should be in the results. Could you describe the process of the interviews?

Figure 1: Could more information be provided? E.g. Positives of what? And add more information to the title?

Patient and public involvement

7 – This section needs rewording? Is this people with ACL rupture involved? Is this just the study authors?

Results:

Table 1: could the scales be provided under the table to help the reader understand what each section means?

Feasibility outcomes

Recruitment rate

- Could this process be described earlier?

Fidelity

- Not clear if this is a single site study? Seems to be multiple.

Table 2 – no need for the disagree collum.

Acceptability

- This manuscript is long – you could remove information available in the table and consider formatting the results in a consistent format to make it easier to follow.

Nested qualitative interviews:

- This section is too long. The information could be condensed and some put into a supplementary file.

Discussion:

- This section needs to be more concise too. E.g. Page 25 17- this information should be reported in the methods.

- I suggest using subheadings and focusing on the feasibility outcomes.

Conclusion:

- This should also focus more on the feasibility findings e.g. how many were met?

VERSION 1 - AUTHOR RESPONSE

Reviewer: 1

A useful study. And exploratory. My main feedback is that I wonder whether the results are presented with too much conviction? For example, is it realistic to say that the intervention was effective in conversations between patients and clinicians? And that the intervention would have been successfully implemented? If so, how was this established?
Thank you for your thorough review of the manuscript. We believe these comments have been addressed in response to specific feedback provided below.

1. Introduction:
    a. Is the last sentence the purpose of the study (This new SDM intervention..... individual qualitative conversations). Perhaps be explicit then?

This has been reworded to clarify the purpose of the study. The sentence now reads:

"The purpose of this study was to explore feasibility, acceptability and indicators of effectiveness of this novel SDM intervention ahead of a potential future main trial."

2. Methods
    a. What were the criteria for purposeful sampling?

Information regarding purposive sampling has been included. The text now reads:
"We planned to interview a purposive sample of six patients (aiming for variability in age, sex, education and health literacy level, response to baseline and follow-up questionnaires) and six physiotherapists (aiming for variability in level of experience, hospital site, number of participants treated in the trial) [10]"

    b. Physiotherapists were also trained. Why was this not mentioned as part of the intervention, since training is different in nature from applying a SDM tool? And was it training or rather an explanation with instruction?

The training was an educational session informing physiotherapists about the trial, the components of the SDM intervention and its delivery in practice. The training on how to deliver the intervention as part of a normal physiotherapy consultation was not part of the intervention.

    c. Can the list of PROs be included in the appendix?

The PROs have been added to the supplementary material (files 4 and 5).

    d. Why were existing instruments not used for the acceptance questionnaire?

An existing instrument to measure the acceptability and meet the aims of the study, as discussed in the preceding study where the intervention was developed with patients and stakeholders, did not exist.

    e. Under Sample size and Recruitment, it states that 6 plus 6 interviews will be conducted. Under Nested Qualitative Interviews, the targets are reduced to 5 plus 5?

The sample size was n=6 for both groups. Recruitment ceased at 5 participants due to data saturation. Additional information has been added to the text to clarify this point, the text now reads:
"A sample of five participants and five physiotherapists were interviewed. As described in the protocol, participants were interviewed until data saturation was reached."

    f. Data collection: Data were mapped on a matrix. Was this conducted by one researcher? Please state the number of researchers and name.

Additional information has been added to the text to clarify this point, the text now reads:
"Data were mapped onto a matrix with five overarching headings (presented above, figure 1) by one author HC."

3. Results:
    a. See my earlier comment: I find the paragraph 'effectiveness' confusing. It suggests that a quantitative effect has been measured and demonstrated. - I also find this paragraph's wording too much of an interpretation instead of the actual results of the interviews. It is stated several times that 'the SDM intervention was perceived to be effective ....'). This fits better under discussion, where then the limitations to this interpretation can be discussed. If the 1st sentence of each paragraph is omitted, the text gains clarity as far as I am concerned.

The heading of the paragraph has been changed to 'indicators of effectiveness' to offer clarity that quantitative effect has not been measured. The first sentence of each paragraph has been omitted as suggested. The following sentences have been amended:

1. "The SDM intervention was perceived to be effective in several areas of practice by both patients and physiotherapists" now reads "The SDM intervention was reported to add value in several areas of practice by both patients and physiotherapists"

2. “The intervention was also perceived to be effective in supporting communication between patients and physiotherapists” now reads “The intervention was also described to support communication between patients and physiotherapists”
3. “Physiotherapists viewed the intervention as more effective than usual care. They reported it to have supported "buy in" from patients, who were perceived to have increased knowledge of their injury and treatment options available to them” now reads “Physiotherapists further reported the intervention to have supported "buy in" from patients, who were perceived to have increased knowledge of their injury and treatment options available to them”
4. “this was also mirrored by patients who reported the interventions effectiveness in providing them with access to healthcare language” now reads ”This was also mirrored by patients who reported the interventions value in providing them with access to healthcare language.”
5. “In addition, physiotherapists reported the effectiveness of the intervention in empowering patients to be involved in their care” now reads “In addition, physiotherapists described the role of the intervention in empowering patients to be involved in their care”

4. Discussion:
    a. The discussion is too long. This does not properly convey the core message. Based on the study aim, I would select some key findings for the discussion and mirror them on previous research and what the next steps could be.
    b. The results are encouraging and legitimate further research and implementation. I would better balance that positive side with modesty.
    c. To make it more to-the-point, I would discuss all the limitations of the research only under this paragraph and not in the text beyond it.
    d. This could also be better reflected in the conclusion: what can practitioners do with these results and, what steps are still needed to really establish applicability and added value for patients and practice?

In response to this and reviewer 2s comments about the discussion, this has been shortened and reworded to offer clarity. The discussion is separated into 3 sections which have been reordered to ensure consistency with the previously stated aim: (1) Feasibility and acceptability, (2) indicators of effectiveness and (3) Strengths, limitations & reflexive considerations. The amendments to the discussion have been copied below to also incorporate reviewer 2’s responses, please see page 11 onwards.

Reviewer: 2

Mr. Andrew Gamble, The University of Sydney
Comments to the Author:
I thank the authors for the opportunity to review this paper. Below are my comments:

1. Abstract:
   If word count allows, is it possible to consider adding the following information?
   Design: could this be more specific?
   Participants: How was ACL rupture determined? MRI? Were they acute ACL rupture?
   Outcomes: How was acceptability measured? Was this via nested qualitative interviews?
   Results: How many participants were contacted? Did you include consent rate? How many were interviewed?

Further information has been added to address these points whilst maintaining the word limit. Further detail cannot be added as the word limit is now at 298 words. The abstract now reads:
“Objectives

To understand feasibility, acceptability and indicators of effectiveness of a shared decision-making (SDM) intervention with patients following an anterior cruciate ligament (ACL) rupture in a UK NHS setting.

Design
Non-randomised feasibility study with embedded qualitative interviews.

Setting
Orthopaedic and Physiotherapy service at an acute NHS Teaching Hospital in the Midlands, UK, between 29 January and 5 June 2024.

Participants
20 patients diagnosed with an ACL rupture following magnetic resonance imaging.

Intervention
An SDM intervention comprising of two components: (1) patient information leaflet, (2) option grid.

Outcome Measures
The primary outcome was to determine feasibility for a definitive trial using four outcomes: (1) recruitment rate, (2) fidelity of intervention delivery, (3) acceptability, (4) follow-up questionnaire completion. The secondary outcome was to explore indicators of the intervention's effectiveness using quantitative data from patient reported outcome measures (acceptability questionnaire and satisfaction with decision scale) and qualitative data from patient and clinician interviews.

Results
20 participants were recruited, mean age 32.2 (SD 9.7), 40% female. Recruitment rate was 95.2%, fidelity of intervention 100%, acceptability 94% and follow-up questionnaire completion 100%. The mean overall satisfaction with decision scale score was 24.85/30 (SD 3.82). There were no adverse events. Data from qualitative interviews with patients (n=5) and physiotherapists (n=5) suggested the SDM was acceptable and appeared effective in: (1) supporting decision-making about treatment, (2) conversations between patients and clinicians, (3) improving patient knowledge, (4) providing patients with access to health language and (5) supporting patients to ask questions deemed important to them.

Conclusion
The novel SDM intervention is acceptable to both patients and physiotherapists. Indicators of effectiveness explored through quantitative and qualitative data suggest the intervention to be beneficial to decision-making processes for patients and clinicians deciding on treatment following an ACL rupture. A full trial is feasible to run in the NHS."

2. Introduction:
    8 – It may be helpful to clarify if you are referring to acute ACL rupture when referring to the RCT conducted as this can change the interpretation of surgical vs non-surgical outcomes. You could say that non-surgical management is a viable option for acute ACL rupture based on two randomised controlled trials to show the need for a shared decision-making tool.

The three RCTs referred to cover both acute and non-acute ACL ruptures, this information has been added and the text now reads:
"Three randomised controlled trials have compared surgical and non-surgical management for patients following acute and non-acute ACL rupture and report conflicting findings [3–5]."

    8- It would be better to use the term 'rupture' or 'injury' throughout

Injury has been replaced with rupture throughout the manuscript.

    12 - How many participants were in the 'UK qualitative study'?

Sentence now reads: “A UK qualitative study (n=18)…”

30 – The beginning of this paragraph focuses on the development process but does not provide the reader with enough information to understand what the shared decision-making tool being evaluated involves.

The content of the shared decision-making intervention is described under Methods on page 7 and a reference is included in this paragraph to the publication detailing the design and content of the intervention. In attempt to keep the manuscript concise, we feel describing this further in the introduction in not necessary.

3. Method:

16 – This section is hard to follow. Can it be restructured to clearly state the aims of this paper compared to those described elsewhere or that will be addressed in the future?

The sentence has been removed to avoid confusion. The statement aimed to offer clarity for the reader that an aim included in the protocol had not been missed but described in a separate publication. The aim of the study has been added to ensure clarity. The text now reads:

“A full description of the methods has previously been published [12]. A brief description is detailed below. There were no changes or deviations from the protocol.

Study Aim

The objective of this study was to understand (1) feasibility, (2) acceptability and (3) indicators of effectiveness, of a SDM intervention with patients following an ACL rupture in a UK NHS setting.”

29 – Is this a single centre study? You say across three sites here?

The study ran across three hospitals which form part of one acute NHS Hospital Trust. As defined by the study sponsor, although this is three hospitals, it is one single site. This has been amended in the text which now reads “Participants were recruited between 29 January 2024 and 5 June 2024 from the orthopaedic and/or outpatient musculoskeletal physiotherapy services at one large acute NHS teaching hospital trust, across three hospitals.”

34 – In the abstract you use the term clinician and here physiotherapist – are all interviews with physiotherapists?

All interviews were conducted with Physiotherapists, wording has been updated throughout the manuscript to replace clinician with physiotherapists where only physiotherapists are being referred to.

36 – What are the eligibility criteria? Is this adults only? Was it within a certain timeframe from ACL rupture? Maybe add this in a box? Or report this before saying they were invited to participate? And how were they invited? What was the process? Who contacted patients at what stage? How? Etc.

Further information has been added to address these comments with the eligibility criteria removed from the text and added to a Figure. The text now reads:

“Potential participants were identified by the orthopaedic team during the patient’s clinical appointment where they were diagnosed with an ACL rupture. Clinical staff also identified appropriate patients from orthopaedic and physiotherapy clinic/waiting lists. They confirmed eligibility (shown in Table 1) and gained consent form the researcher to make contact with the patient to discuss the study. On confirmation the potential participant met the eligibility criteria, they were invited to participate in the study and provided with the participant information sheet (PIS) and consent documents.”

**Inclusion criteria**

• Age 18 or over<br>• First-time ACL rupture (in that limb) confirmed by a magnetic resonance imaging (MRI) scan
**Exclusion Criteria**
• Concomitant injury requiring surgical intervention anticipated to significantly alter usual treatment e.g. fracture, bucket handle meniscal tear requiring immediate surgical intervention prior to ACLR<br>• Previous surgery to the affected limb<br>• Pregnancy (as this is likely to affect decision-making regarding surgical treatment and rehabilitation)

Table 1 – Eligibility Criteria"

a. Data collection:

3 – what were the questionnaires? Is this referring to baseline only?

This refers to the follow-up questionnaire outlined in the above paragraphs, the text has been reworded to ensure clarity and now reads: "Participants were given the choice of receiving the follow-up questionnaires (SWD and acceptability questionnaire) online"

Intervention

17 – Could you include what evidence the SDM tool was based on?

As per the previous response, the design and content of the intervention is detailed in a separate publication. In attempt to keep the manuscript concise, we feel describing this further is not necessary.

21 – What are the questions about?

The reader has been directed to the supplementary material for information regarding the questions. The text now reads "The option grid was a 2-sided A4 page directly comparing surgical with non-surgical treatment in response to nine key questions (shown in supplementary file 3)."

27 – A link was provide? Were they given a physical copy?

The text has been amended and now reads: "Participants were able to access the intervention via a physical paper copy and/or digitally via pdf documents. The paper and/or digital intervention was provided to patients on consenting to take part in the feasibility study (according their participant preference)."

28 – What does 'digital intervention' refer to? Is this viewing the SDM tool? Or questions mentioned previously? This isn't clear.

The text has been amended to offer clarity relating to this point, the sentence now reads: "Participants were able to access the intervention via a physical paper copy and/or digitally via pdf documents. The paper and/or digital intervention was provided to patients on consenting to take part in the feasibility study (according to participant preference). The digital copy was delivered via the study website (www.pop-aclr.co.uk) on a password protected page allowing patients to download pdf versions of the paper documents. The intervention was subsequently discussed with patients at an outpatient appointment with a trained Physiotherapist."

35 – There needs to be more information about the physiotherapists involved in the trial earlier or more consistency with terminology used. Was it an objective to evaluate the training of physiotherapists involved in the trial too?

There are too many terms used to describe the 'patient information leaflet' digital intervention' etc.

Wording has been updated throughout the manuscript to replace clinician with physiotherapist to ensure clarity on this point (in line with a previous comment). It was not an objective to evaluate training of physiotherapists involved in the trial. Wording has been amended throughout this section in line with this suggestion.

Feasibility

9 - This section is clearer but needs to match the information and terminology used prior.

Thank you for highlighting the inconsistency in terminology. This has been amended to ensure consistency through the manuscript.

20 – was acceptability questionnaires given after viewing the SDM tool or after consultation with the physiotherapist?

This has been amended to clarify this point. The text now reads: "(3) Acceptability was measured using both quantitative and qualitative data. All patients participating in the study were asked to complete an acceptability questionnaire after the scheduled consultation with the physiotherapist."

42 This could be reworded to avoid confusion that this was feedback about the SDM tool.

Thank you for the suggestion. This has been reworded and now reads: "Feedback on the acceptability statements was therefore gained from clinicians, a therapy manager, patients and academic experts."

b. Nested qualitative interviews:

- The number interviewed should be in the results. Could you describe the process of the interviews?

When reporting the participant interview sample, it is typical that this is reported in the methods section of the manuscript, this is in line with the SRQR checklist which is included in supplementary file 2. Further information regarding the interviews is included in the results which reads:

"**Nested Qualitative Interviews**

Individual semi-structured interviews were completed with five patient participants and five physiotherapist participants. Demographic data of the patient and physiotherapist participants is shown in supplementary file 7."

Figure 1: Could more information be provided? E.g. Positives of what? And add more information to the title?

Further information has been added to Figure 1 and its title. Figure 1 is now displayed as:

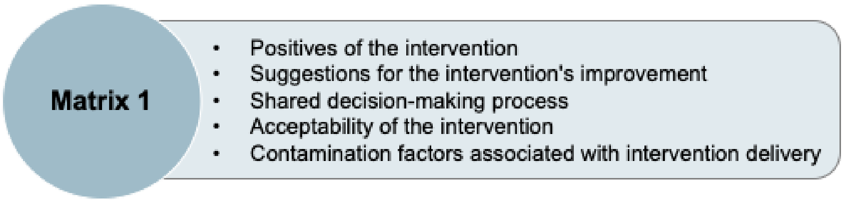

Figure 1 - Data matrix guiding qualitative interview data collection and analysis

c. Patient and public involvement

7 – This section needs rewording? Is this people with ACL rupture involved? Is this just the study authors?

People with experience of an ACL rupture were involved. PPI members were not the study authors. The text has been reworded to offer clarity on this point & now reads: "Patients and

key stakeholders (including orthopaedic surgeons, therapy managers and therapists) with experience of sustaining an ACL rupture, treating patients following rupture or managing departments where patients seek treatment following an ACL rupture were involved in the grant funding application, study design and set up.”

4. Results:
   Table 1: could the scales be provided under the table to help the reader understand what each section means?\

The scales are provided under Table 2 (amended due to the addition of a table earlier in the manuscript) which now reads:
“*Index of Multiple Deprivation Decile represents ten equal sized groups from one (most deprived 10% of areas nationally) to ten (least deprived 10% of areas nationally).
†Level of education is reported from no formal qualification to level 8, examples of qualifications at each level include: Level 1 GCSE grades D-G or 3-1, Level 2 GCSE grades A*-C or 9-4, Level 3 A level, Level 4 certificate of higher education, Level 5 diploma of higher education, Level 6 degree with honours, Level 7 master’s degree, Level 8 doctorate.
±REALM-R scores are reported from 0-8, a score of 6 or below identifies an individual to be at risk of poor health literacy.”

   a. Feasibility outcomes
      Recruitment rate
      - Could this process be described earlier?

This paragraph describes the results for recruitment of participants into the study. They have been described in the method section, and we feel the results are appropriately placed here.

   b. Fidelity
      - Not clear if this is a single site study? Seems to be multiple.

In response to a previous comment, detail has been added to explain the hospitals and single site setup. This sentence has been reworded to avoid confusion and now reads: “All physiotherapists completed the case report form in full and indicated 100% intervention fidelity. There were 11 physiotherapists who delivered the intervention.”

   c. Table 2 – no need for the disagree collum.

The response to disagree was desired for some statements e.g. “The presentation of the information seemed in favour of not having surgery”. We feel it is important to include both columns to offer clarity for the reader where the overall agreement percentage has been taken from.

   d. Acceptability
      - This manuscript is long – you could remove information available in the table and consider formatting the results in a consistent format to make it easier to follow.

This section has been reformatted with information removed to ensure consistency. This section now reads;
“Acceptability
The overall level of acceptability was 93.67% as reported above. Acceptability was also explored during individual qualitative interviews (discussed below).”

   e. Nested qualitative interviews:
      - This section is too long. The information could be condensed and some put into a supplementary file.

This section has been condensed in addition to placing all previously included quotes in a supplementary file (file 10). This section now reads:
“Nested Qualitative Interviews

Individual semi-structured interviews were completed with five patient participants and five physiotherapy participants. Demographic data of the patient and physiotherapy participants is shown in supplementary file 7.

Interview data are described in relation to three themes exploring: (1) acceptability of the intervention, (2) indicators of effectiveness, and (3) feedback of trial procedures.

Theme 1: Acceptability

The SDM intervention was positively received by patients and physiotherapists and overall deemed to be acceptable with respect to its content and delivery.

Patients reflected positively on the intervention's length, colour scheme, font size, level of detail, medical language and lay terminology used, paper quality and availability of the paper and online version. Participants suggested that the intervention struck an appropriate balance of offering relevant information without excessive, lengthy and confusing medical detail (Quote 1, Supplementary File 10).

Two participants explained to be disappointed by some data as most expected the outcomes of surgery to be higher than that reported in the literature. However, they went on to reflect positively about their inclusion as it offered transparency of outcomes for the available treatment options (Quote 2, Supplementary File 10).

Both patients and physiotherapists reflected positively on timing of intervention delivery, with patients highlighting the value of its provision at the point of diagnosis, followed by an appointment where it was expected to be discussed. Patients reported that this allowed time to process the information and consider relevant questions. All participants felt the physiotherapist was an appropriate healthcare professional to be discussing decision-making about treatment with (Quote 3, Supplementary File 10).

One patient reflected on the importance of the healthcare professional to be interested in the area and not "just going through the motions" [0012, patient], with another commenting on their preference for delivery by a "knee specialist" [0009, patient]. One participant suggested a one-to-one appointment may not be necessary. They felt the SDM intervention contained sufficient detail to support decision-making about treatment, particularly where ACLR was seen to be inevitable for young patients looking to return to high level cutting and pivoting sports.

Physiotherapists reported the intervention to be acceptable describing it to be non-burdensome, compatible with usual practice, low risk and within the scope of a qualified Physiotherapists' skillset. The intervention was low/minimal cost to participants apart from the time taken to engage with it which was reported to be acceptable. One therapist reflected upon the increased time of a 60-minute appointment allowing for more in-depth conversations, which was preferred to 30-minutes. In contrast, some physiotherapists felt the intervention supported a reduction in consultation time.

Theme 2: Indicators of Effectiveness

The SDM intervention was reported to add value in several areas of practice by both patients and physiotherapists. Patients described the intervention to increase their knowledge of the injury and the treatment options available to them which supported them to engage in decision-making (Quote 4, Supplementary File 10).

In contrast, one participant reflected on the lack of compelling evidence available and reported in the SDM intervention and suggested this added difficulty to the decision-making process (Quote 5, Supplementary File 10).

Inclusion of the statistical data was seen to add value. It was viewed as evidence-based and relevant to what happens in real life. Several patients commented on use of statistics to support an individual assessment of the pros and cons of treatment, in line with what was important to them (Quote 6, Supplementary File 10).

Physiotherapists also reported the level of detail, statistical data and information included in the intervention to be appropriate, relevant and useful. They reflected that, without the intervention, they would not have been able to offer the same level of evidence-based information as keeping up-to-date with the literature is challenging in the current NHS climate (Quote 7, Supplementary File 10).

The intervention was also described to support communication between patients and physiotherapists. Further, all patients reported to use the intervention outside of the trial, with friends, family members, healthcare professionals and employers to help communicate their injury and gain support to manage its consequences. Both physiotherapists and patients deemed the intervention to be trustworthy as it was derived from research and included references to the literature that were easy to locate should participants want to seek further information. As a result, some patients suggested to have not needed to conduct internet research to understand their injury further, saving them time from reading (or watching) unwanted levels of detail (Quote 8, Supplementary File 10).

Physiotherapists further reported the intervention to have supported "buy in" from patients, who were perceived to have increased knowledge of their injury and treatment options available to them. They felt the intervention offered a good foundation level of knowledge for patients which supported a more pragmatic appointment that was structured, streamlined and tailored to each individual (Quote 9, Supplementary File 10).

This was also mirrored by patients who reported the interventions value in providing them with access to healthcare language. Patients reflected on their ‘non-medical background’ and explained that the intervention increased their understanding of appropriate terminology, allowing them to have individualised conversations with healthcare professionals and ask important questions relevant to them (Quote 10, Supplementary File 10).

In addition, physiotherapists described the role of the intervention in empowering patients to be involved in their care. They felt this enabled a greater sense of control and improved patients’ motivation as they were perceived to have higher accountability for the treatment and its outcomes. They reflected on the interventions presentation of information and felt it was fair and balanced towards both treatment options, ensuring it was effective for use with all patients following an ACL rupture. Physiotherapists further described the interventions utility in supporting their skills to deliver evidence-based information and have shared decision-making conversations with patients. This was reflected by physiotherapists in relation to patients following an ACL rupture, but also for patients with other musculoskeletal/orthopaedic conditions as part of usual practice as opposed to RCT type practice. They explained to feel more confident to take part in SDM and explore patient views about treatment (Quote 11, Supplementary File 10).

Theme 3: Trial Procedures

Both patients and physiotherapists had a positive attitude towards participating in the feasibility study. Patients reflected on the low burden of engagement with the intervention, completing baseline data collection and PROs electronically in-person or via mobile and email links.

Three areas identified by physiotherapists for future trial refinement included, use of drop-in sessions for training refreshers (although not favoured by all), a QR code to access the clinician resources page on the website (replacing access via a password which was or could be forgotten) and a reminder/checklist of key intervention components to be embedded within a patient’s electronic record. Physiotherapists reported the use of a ‘study champion’ and

availability to email and receive a prompt response from a member of the research team to have been successful during the feasibility study and should be incorporated into a future main trial to support delivery.

Future contamination factors were also explored with physiotherapists. When asked about delivery of usual care in parallel to the SDM intervention (as part of an effectiveness trial), they expressed perceived difficulty with doing so given the new knowledge obtained from the SDM intervention. They favoured a design where treatment would be delivered for one intervention arm only. This is an important consideration and reflection given some therapists had also already begun using the intervention, or elements of it, in their usual practice.

Shared Decision-Making Intervention Refinements
As a result of participant feedback during interviews, the following refinements were suggested: (1) additional information to be added about the use of braces for non-surgical management of ACL ruptures, and where to purchase from (2) a prompt for clinicians to review MRI scan results with patients, (3) greater detail about physiotherapy treatment, (4) signposting for local mental health services, (5) additional detail about the surgical procedure and potential additional procedures e.g. tenodesis, (6) adding an arm to the spider diagram to prompt patients to ask 'will I need to stop taking my other medication', (7) addition of statistical data reporting patient satisfaction following surgical or non-surgical treatment. One participant suggested deleting the information about ACL self-healing as there was not enough research to support its inclusion.

These suggestions were discussed at TMG meetings to support refinement of the intervention and its logic model (Implementation Research Logic Model, IRLM). It was agreed that the following components were added as they were deemed to add value to the intervention whilst ensuring it remained concise and maintained the principles and design of the original content. The following amendments were made: (1) a prompt for signposting to local mental health services was added to the information about psychological factors (option grid, 'what else can I do?', page 2), (2) the question 'will I need to stop taking my medication?' was added under the question 'how will treatment impact my life? (patient information leaflet, spider diagram, page 8) and (3) satisfaction with treatment data was added (patient information leaflet, page 5). One participant identified a typo in the option grid which was also corrected.

The final version of the SDM intervention (patient information leaflet and option grid) is presented in supplementary file 8, with refinements in green text.

The IRLM was also revised and is presented in supplementary file 9, with refinements in green text. These refinements were also supported by implementation factors identified as barriers and enablers to future normalisation, described in a separate publication."

5. Discussion:
   - This section needs to be more concise too. E.g. Page 25 17- this information should be reported in the methods.

We have added this information to the methods to which reads:
"Interviews were conducted by the lead researcher (HC) who is also a physiotherapist working at UHDB."

The information about the author conducting the qualitative interviews is also included here as a reflexive consideration and potential limitation. On balance with the previous reviewers' comments ensuring all limitations are reported in this section, we have left the information in this section but reduced the overall word count in aim of being more concise. Amended discussion copied below.

   - I suggest using subheadings and focusing on the feasibility outcomes.

Subheadings have been added to the discussion with the feasibility outcomes appearing first to ensure focus to these outcomes.

The text has been amended to ensure concise information that reflects the results in response to yours and reviewer 1's comments. We have also condensed the text to reduce the length of the manuscript. The discussion now reads:

"DISCUSSION

The results of this study confirm that the intervention is feasible to deliver and acceptable to both patients and physiotherapists. Indicators of effectiveness suggest the interventions potential utility in supporting SDM processes for treatment decision-making following an ACL rupture. A full trial exploring the effectiveness of the SDM intervention is feasible.

Feasibility and acceptability

All four feasibility outcomes were achieved between 94-100%.

Recruitment rate

The overall recruitment rate of 95.2% is encouraging and suggests patients are willing to consent to participate in a trial of this nature. During qualitative interviews, patients and physiotherapists reflected on the low burden of the intervention, timing at which it was delivered and appropriateness for a wide scope of patients following ACL rupture as eligibility criteria allowed for inclusion of participants with both acute and chronic ACL ruptures aged 18 and older. These factors likely aided recruitment and so a similar strategy in a future trial is warranted. It is however, important to highlight the high recruitment rate in the context of other musculoskeletal and orthopaedic research which is typically less than 50% ([19,20]). The 6-month recruitment window was sufficient to recruit 20 patients at one NHS Trust (with recruitment achieved ahead of schedule within four-and-a-half-months) and so a similar modelling strategy can be used in a future trial at sites with similar ACLR rates.

Fidelity

Fidelity of intervention delivery was 100% for all 11 physiotherapists involved in the study. Ahead of a participants' appointment, physiotherapists' were sent an email reminder of key procedures, likely contributing to high fidelity to the intervention. Fidelity was assessed via a self-reported checklist by the treating physiotherapist; however, this was not externally verified for accuracy. Observation of consultations in a future study would offer clarity regarding fidelity of delivery.

Acceptability

Overall acceptability was 93.67% across the 15 items in the acceptability questionnaire. A recent study exploring patient acceptability of a decision aid following Achilles tendon rupture (n=13) reported similar positive responses amongst patients regarding acceptability [21]. This suggests patients following an MSK/orthopaedic injury respond well to interventions supporting SDM and demonstrates appetite for future research in this area. A key perquisite to decision-making and essential to SDM is to ensure that patients are aware of the treatment options available to them [22]. In this study, 100% of patients felt 'the presentation of information was 'balanced' and fair towards each treatment option'. However, the lowest scoring question at 80% was that 'the presentation of information seemed in favour of having surgery' which conflicts the previous response. Interviews with patients revealed that although participants viewed the wording to be balanced, they felt research available to support a decision to proceed non-operatively was lacking. In addition, some participants described consultations which lacked clinical equipoise and leaving patients uncertain about the validity of non-operative treatment. Similar to the previously discussed point, observation of consultations in a future trial may offer greater depth to the presentation of equipoise and impacts upon SDM.

Follow-up questionnaire completion

Follow-up outcome measure completion was 100% with acceptability of online methods via text and email links. Participants received a £20 voucher of their choice on receipt of questionnaire responses which may have contributed to the high response rate. During patient interviews, participants stated that the questionnaires were not burdensome, were relevant and easy to complete following the hyperlinks sent via text or email.

Only one patient failed to attend their pre-booked physiotherapy appointment, however, they did not withdraw from the trial and proceeded to complete the follow-up questionnaires. They were invited to interview to explore this further however did not respond. During qualitative interviews with other participants, some suggested the face-to-face interaction may not be necessary for all, as they felt the SDM intervention covered sufficient information to support decision making about treatment (supplemented by discussions with the orthopaedic clinical team). Some patient participants valued the one-to-one consultation with the physiotherapist and felt the opportunity to ask personalised questions arising from reading the SDM intervention was important. Ensuring the one-to-one support patients receive alongside the SDM intervention is tailored to individual needs, would be an important element of a future trial and may offer more pragmatic approach to engaging with SDM.

Indicators of Effectiveness

Although it was not the focus of the feasibility study to determine effectiveness (nor was it powered to do so), the SWD scale was used to explore indicators of the intervention's effect on decision-making processes. The average SWD score was 24.85 (95% CI 23.06-26.64, SD 3.82). Only one participant disagreed with one statement, '(that they were adequately informed). This could not be explored further as the participant did not respond to the invitation to interview. Interviews with another participant, who indicated to 'neither agree nor disagree' with the same statement suggested the reason for this to be because they felt there was not enough information available (in the literature) to be adequately informed about the non-operative treatment pathway (as discussed above). This could be one reason for the participant disagreeing with the statement. Another could be that the intervention was not effective in improving knowledge for patients to consider that they were adequately informed. There was however evidence from both patient and physiotherapy interviews that the intervention was useful in supporting improvements in knowledge. As this study was with a small number of participants, this will be an important avenue to consider in a future larger trial to understand potential unmet needs. Another consideration is that the follow-up questionnaire was completed too soon after interaction with the intervention and patients may have needed greater time to consider its impact.

Patients reflected on the interventions utility in providing them with access to healthcare language and the subsequent ability to ask questions deemed relevant to them. NHS England outline current literacy levels in the UK as a crisis impacting upon people's health [29]. In England, a 2018 survey revealed 16.4% of the population to have poor literacy skills, defined as reading at or below the level of a 9-year-old [30]. A further 43% are thought to not understand written health information, with this rising to 60% when numbers and statistics are included [29,31]. The inclusion of statistical data was viewed favourably by both patients and physiotherapists and there was no significant difference in outcomes between those identified to be at risk of poor health literacy and those who were not. Assessing participants health literacy is therefore an important element of a future trial to further understand the utility of the intervention in those with lower levels of education and at risk of or deemed to have poor health literacy skills.

There was some evidence of uncertainty with decision-making about treatment. One participant reported difficulty with the lack of clarity offered by the statistical data included in the intervention and discussed the challenge of making an informed decision. Uncertainty with decision-making in usual care following an ACL rupture has previously been reported [6]. Uncertainty with the non-operative treatment pathway has also previously been reported in an

individual interview study with Scandinavian patients undergoing non-operative treatment following ACL rupture [32]. It is unknown how the participant in this feasibility study would have experienced the usual care pathway and whether the intervention had no effect on or added to existing uncertainty or whether it may have introduced it. Evidence from other patients suggested that the intervention did not introduce doubt, but instead offered transparency to the treatment pathway which they reflected positively on. A longer time frame to complete the follow-up questionnaires may add greater understanding to this phenomenon in a future trial. Although previous research has identified most patients value the opportunity to be involved in decision-making, some may require more support and greater time for this process than others [33,34]. The patient demonstrating uncertainty with decision-making in this study, identified that they were likely to have responded differently (and more positively) to the follow-up questionnaires (acceptability and SWD) had they have been given more time to engage with the intervention before appraising it. Participation in decision-making has been associated with improved health outcomes and overall greater satisfaction with the consultation process [35,36]. Understanding change in SWD scores over time and in relation to specific points of interaction on the patient pathway may therefore be important components of a future trial.

In addition, most participants expected the outcomes of ACLR to be better than that reported in the intervention. This expectation has previously been reported in an Australian cohort study, where only 24% of patients had returned to their pre-injury level of sport at 1-year despite 91% expecting to when asked preoperatively [37]. Patients' preoperative expectations of ACLR have also been linked to motivation to engage in rehabilitation and overall treatment satisfaction [38–40]. A predictors review completed in 2023, identified patients' positive estimation of their ability to return as a predictor of a successful return to physical activity [41]. However, it was highlighted that clinicians should take caution to ensure patients' expectations are not enhanced above levels that are realistic. This is an important role of the SDM intervention, with evidence from this study that engagement with it offered a pragmatic view of treatment outcomes (predominantly a return to physical activity) that would have otherwise been assumed to be higher.

Elwyn et al's. study also reported an average time of 5 minutes and 20 seconds to cover the content in their option grid [7]. Despite this, overall encounter time was no different when using the option grid compared to consultations where the option grid was not used. Whilst time taken to discuss the SDM intervention was not recorded, reports from both patients and physiotherapists confirmed low burden and no perceived negative effects to consultation time. One therapist with experience of a 30- and 60-minute consultation acknowledged that whilst 60-minutes was preferred, delivery was achievable within 30 minutes. This suggests that the intervention was appropriate for use during usual clinical practice within a physiotherapy consultation. Clinician time constraints have previously been identified as a major barrier to implementing SDM [42]. This was reported by one patient participant who felt SDM was not prioritised by the physiotherapist due to time constraints. Exploration in a future full trial would confirm the interventions effectiveness in supporting SDM processes, how it is prioritised by clinicians, and its impact on consultation length.

Strengths, Limitations and Reflexive Considerations

Data were collected on participants' ethnicity. Capturing ethnicity data is recognised in the 2022 (most recent) UK NLR report as a future priority to support understanding of the epidemiology and outcomes following ACL rupture [43]. Whilst we acknowledge the present study included only 20 participants in one area of the UK, capturing this data may support future understanding of the demographics of those with ACL ruptures. Without consistent reporting of ethnic background in the literature, it is unclear whether the findings of this study are transferable to the wider UK ACL rupture population.

A second strength of this study is that participants' level of deprivation, level of education and health literacy were recorded, to support understanding of the SDM interventions utility in those at risk of poorer healthcare experiences and outcomes. Whilst no significant differences were detected between groups, this only offers an indication of the intervention's utility in SDM process as the study was not powered to detect effectiveness. This is an important area of exploration in a future trial to ensure individuals from lower socioeconomic backgrounds are not at risk of marginalisation from SDM processes.

The population further included those from a wide age range, with varying levels of pre-injury and current physical activity, who sustained their ACL rupture via contact and non-contact mechanisms. Although this was only a small number of participants, these demographics would suggest that the study captured an appropriate sample encompassing a range of characteristics representative of the reported adult ACL population in the current literature [9].

Although there were provisions in place to recruit non-English speaking participants, none were identified as eligible for the trial. Future consideration to understand how the material may translate into other languages and its utility with non-English speaking participants may therefore be needed. All participants identified to be at risk of poor health literacy declined to be contacted to share their experiences in a qualitative interview. We therefore may not have captured a full range of perceptions and experiences, particularly of those at risk of poor health literacy.

Further, the qualitative interviews were carried out by the lead researcher who also identifies as a clinician at one of the physiotherapy departments running the study and who has previously practiced at the second department. The lead researcher has been a member of the clinical team for several years. This may have limited the discussions with physiotherapists who may not have felt comfortable accounting negative experiences to a clinical colleague."

6. Conclusion:
   - This should also focus more on the feasibility findings e.g. how many were met?

The conclusion has been reworded to focus on the feasibility findings, this now reads: "All four feasibility outcomes were achieved between 94 and 100%. This study has demonstrated that implementation of an SDM intervention in the ACL rupture pathway is acceptable to both patients and physiotherapists. Indicators of effectiveness have been identified and reflect on the interventions utility in improving patient knowledge, supporting patients to understand medical terminology to formulate clinically relevant questions, facilitating SDM conversations, empowering patients to be involved in decision-making about their treatment and providing up-to-date clinically relevant information for both patients and clinicians."

VERSION 2 - REVIEW

**Reviewer**	**1**
**Name**	**van Veenendaal , Haske**
**Affiliation**	**Erasmus University Rotterdam, ESHPM**
**Date**	**03-Jul-2025**
**COI**	

Thank you for carefully addressing and processing the comments on the first version of the manuscript, which, in my opinion, has led to an improved version. I still have a few suggestions:

In particular, I would look at the discussion, that is till too extensive, to further shorten it and focus more on the core message. Here are some examples:

- I would include "All four feasibility outcomes were achieved between 94-100%" after the first sentence, as it now appears somewhat lost a few lines lower.

- "achieved ahead of schedule within four-and-a-half-months, in the recruitment rate paragraph could be added after 'The overall recruitment rate of 95.2%.' Then the last sentence can be omitted.

- I am struggling to understand why the follow-up questionnaire completion is discussed in the discussion. There were four main feasibility outcomes: (1) recruitment rate, (2) fidelity, (3) acceptability, and (4) follow-up questionnaire completion. So (3) pertains to the content of the list and (4) is about whether people completed the list? And this is reported in Table 3 in the results section? Why is this considered part of feasibility? It says something about the compliance in providing their PROs, but it is ultimately not part of the intervention to be implemented? And only the first paragraph ('Follow-up outcome measure completion … following the hyperlinks sent via text or email') pertains to the questionnaire completion? Consequently, the paragraph 'Only one patient failed … to engaging with SDM' can be removed.

- I would try to halve the size of the paragraph Indicators of Effectiveness.

**Reviewer**	**2**
**Name**	**Gamble, Andrew**
**Affiliation**	**The University of Sydney**
**Date**	**07-Jul-2025**
**COI**	

I thank the authors for taking the time to make the edits to this paper and thank you for the opportunity to review the response. The changes have improved the manuscript but I do feel that some areas need further consideration. Below are some specific suggestions.

Reviewer 2:

Abstract:

Objectives:

To understand feasibility, acceptability and indicators of effectiveness of a shared decision making (SDM) intervention with patients following an anterior cruciate ligament (ACL) rupture in a UK NHS setting.

- You could remove 'in a UK NHS setting' as this is stated below in Setting to help with word limit and try to avoid abbreviations.

Participants

- Remove '20' as this is a result.

Intervention

An SDM

- 'Delivery of a shared decision making education leaflet including an option grid by..'?

Outcome measures:

'determine feasibility for a definitive trial'

- Is this more appropriate for 'objectives'?

- You could format as 'Primary outcomes: 1) recruitment rate, …' for both primary and secondary outcomes to reduce word count if needed.

Results:

- Ideally you could report the number of participants screened and who participated.

- I would number each outcome in the same format as the outcome section for primary outcomes as now it is confusing that the numbers are used for the secondary outcomes.

Conclusion

- I would try to match the use of terms feasibility, acceptability and indicators of effectiveness to outcomes and try to keep this consistent from the objectives.

Introduction:

- Perhaps you could still use a brief term as per the abstract to help readers know what the tool is?

Methods

- Thank you for addressing the comments in detail. It reads much better now.

Results:

- Make sure to use the full headings e.g. 'Acceptability of the intervention'

- This section is much improved but could still be more concise.

- There are too many paragraphs, and it is difficult to see the structure. For example, you could use paragraphs to separate patient vs physiotherapist views.

Discussion

- The subheadings make it much easier to follow but make sure to be consistent with heading vs subheadings formatting – you may need more subheadings following the feasibility headings or 'indicators of effectiveness' is much longer?

- At times some points are not specific enough. E.g. 'between 94-100%' may need clarification of what this means or removed.

- There are some good points in the discussion, but the structure still needs to be refined.

Strengths and Limitations

- I would suggest using the full term 'shared decision making' rather than 'SDM" here

- Some of the point could be more concise. For example. 'Qualitative interviews with patients and clinicians provided deeper exploration of feasibility, acceptability and indicators of effect of the intervention'

Conclusion:

- Between 94-100% of what?

- The conclusion could still better match the objectives and focus on what the main findings.

- E.g. Indicators of effectiveness showed the shared decision making tool… based on…

- Maybe finish with a stronger statement on why shared decision making is needed in ACL decision making and the next step identified/planned?

VERSION 2 - AUTHOR RESPONSE

Reviewer: 1

Dr. Haske van Veenendaal , Erasmus University Rotterdam

Thank you for carefully addressing and processing the comments on the first version of the manuscript, which, in my opinion, has led to an improved version. I still have a few suggestions:

In particular, I would look at the discussion, that is till too extensive, to further shorten it and focus more on the core message. Here are some examples:
I would include "All four feasibility outcomes were achieved between 94-100%" after the first sentence, as it now appears somewhat lost a few lines lower.
- "achieved ahead of schedule within four-and-a-half-months, in the recruitment rate paragraph could be added after 'The overall recruitment rate of 95.2%.' Then the last sentence can be omitted.

The text has been amended in line with the suggested changes. We have included the revised discussion below (please see page 6 onwards) to address this and reviewer 2's comments.

- I am struggling to understand why the follow-up questionnaire completion is discussed in the discussion. There were four main feasibility outcomes: (1) recruitment rate, (2)

fidelity, (3) acceptability, and (4) follow-up questionnaire completion. So (3) pertains to the content of the list and (4) is about whether people completed the list? And this is reported in Table 3 in the results section? Why is this considered part of feasibility? It says something about the compliance in providing their PROs, but it is ultimately not part of the intervention to be implemented?

Thank you for your comment to clarify this point. While we acknowledge that follow-up questionnaire completion is not part of the intervention itself, it was identified as one of the four feasibility outcomes for this study (alongside recruitment rate, fidelity and acceptability) and was included in the published protocol. Its inclusion is important for assessing whether a future definitive trial is feasible, particularly in terms of a participants' willingness and ability to complete the outcome measures (acceptability questionnaire and SWD scale) necessary for evaluation of the intervention. Therefore, we believe it is appropriate to discuss alongside the other feasibility outcomes in both the results and discussion section to provide a comprehensive overview of the study's feasibility findings. We have structured the discussion in this way to ensure clarity and alignment with the published protocol and to address a related point raised by reviewer 2 (please see below for further detail, page 6 onwards).

And only the first paragraph ('Follow-up outcome measure completion … following the hyperlinks sent via text or email') pertains to the questionnaire completion? Consequently, the paragraph 'Only one patient failed … to engaging with SDM' can be removed.

This paragraph has been deleted.

- I would try to halve the size of the paragraph Indicators of Effectiveness.

This section has been amended in line with this and reviewer 2's additional comments. Please see below for a detailed response (page 6 onwards).

Reviewer: 2

Mr. Andrew Gamble, The University of Sydney

I thank the authors for taking the time to make the edits to this paper and thank you for the opportunity to review the response. The changes have improved the manuscript but I do feel that some areas need further consideration. Below are some specific suggestions.

Abstract:

Objectives:
To understand feasibility, acceptability and indicators of effectiveness of a shared decision making (SDM) intervention with patients following an anterior cruciate ligament (ACL) rupture in a UK NHS setting.
- You could remove 'in a UK NHS setting' as this is stated below in Setting to help with word limit and try to avoid abbreviations.

The text has been amended as suggested and now reads: "To understand feasibility, acceptability and indicators of effectiveness of a shared decision-making (SDM) intervention with patients following an anterior cruciate ligament (ACL) rupture."

Participants
- Remove '20' as this is a result.

This has been removed as per your suggestion.

Intervention
An SDM
- ‘Delivery of a shared decision making education leaflet including an option grid by..’?

The text has been amended and now reads: “Delivery of a shared decision-making intervention which comprised of two components: (1) patient information leaflet, (2) option grid.”

Outcome measures:
‘determine feasibility for a definitive trial’
- Is this more appropriate for ‘objectives’?
- You could format as ‘Primary outcomes: 1) recruitment rate, …’ for both primary and secondary outcomes to reduce word count if needed.

The previous version was formatted in this way, however it was amended at the editors request to remove subheadings.

Results:
- Ideally you could report the number of participants screened and who participated.
- I would number each outcome in the same format as the outcome section for primary outcomes as now it is confusing that the numbers are used for the secondary outcomes.

We have included how many patients were approached to take part in the study and replaced the numbers for the secondary outcomes with letters, this section now reads: ‘21 patients were approached to take part in the study, 20 were recruited with a mean age of 32.2 (SD 9.7), 40% were female. Recruitment rate was 95.2%, fidelity of intervention 100%, acceptability 94% and follow-up questionnaire completion 100%. The mean overall satisfaction with decision scale score was 24.85/30 (SD 3.82). There were no adverse events. Data from qualitative interviews with patients (n=5) and physiotherapists (n=5) suggested the SDM was acceptable and appeared effective in: (a) supporting decision-making about treatment, (b) conversations between patients and clinicians, (c) improving patient knowledge, (d) providing patients with access to health language and (e) supporting patients to ask questions deemed important to them.’

Conclusion
- I would try to match the use of terms feasibility, acceptability and indicators of effectiveness to outcomes and try to keep this consistent from the objectives.

The terms ‘acceptable’, ‘indicators of effectiveness’ and ‘feasible’ have been used in the conclusion. We have amended the wording to add further clarity, this section now reads: ‘The novel SDM intervention is acceptable to both patients and physiotherapists. Indicators of effectiveness explored through quantitative and qualitative data suggest the intervention to be beneficial to decision-making processes for patients and clinicians deciding on treatment following an ACL rupture. All four feasibility outcomes were achieved indicating a full trial is feasible to run in the NHS.’

Introduction:

- Perhaps you could still use a brief term as per the abstract to help readers know what the tool is?

A brief sentence has been added which reads “The intervention, comprising of a patient information leaflet and option grid, aims to ensure patients can make informed decisions about their treatment and that, if chosen, the surgical pathway is appropriate.” In line with our previous response, further detail pertaining to the content of the

intervention is included on page 6 of the manuscript with a reference in the introduction to the publication further detailing the content.

Methods

- Thank you for addressing the comments in detail. It reads much better now.

Results:

- Make sure to use the full headings e.g. 'Acceptability of the intervention'

The headings have been amended to 'Fidelity to the intervention' and 'Acceptability of the intervention'

- This section is much improved but could still be more concise.
- There are too many paragraphs, and it is difficult to see the structure. For example, you could use paragraphs to separate patient vs physiotherapist views.

Thank you for your suggestion. We have revised the structure of the results, reducing the number of paragraphs to improve clarity. The original paragraph structure was laid out in this way to accommodate supporting quotes, now these have been moved to the supplementary material we agree that your suggestion has helped improve the flow of the text. Whilst we considered separating patient and physiotherapist views on initial drafting of the manuscript, we chose to present them concurrently to enhance readability and reflect the integrated nature of the themes. Additionally, separating the perspectives would increase the word count, which we have been mindful to keep concise in response to previous comments. The results now read:
'Individual semi-structured interviews were completed with five patient participants and five physiotherapist participants. Demographic data of the patient and physiotherapist participants is shown in supplementary file 7. Interview data are described in relation to three themes exploring: (1) acceptability of the intervention, (2) indicators of effectiveness, and (3) feedback of trial procedures.

Theme 1: Acceptability of the Intervention
The SDM intervention was positively received by patients and physiotherapists and overall deemed to be acceptable with respect to its content and delivery. Patients reflected positively on the intervention's length, colour scheme, font size, level of detail, medical language and lay terminology used, paper quality and availability of the paper and online version. Participants suggested that the intervention struck an appropriate balance of offering relevant information without excessive, lengthy and confusing medical detail (Quote 1, Supplementary File 8). Two participants explained to be disappointed by some data as most expected the outcomes of surgery to be higher than that reported in the literature. However, they went on to reflect positively about their inclusion as it offered transparency of outcomes for the available treatment options (Quote 2, Supplementary File 8).

Both patients and physiotherapists reflected positively on timing of intervention delivery, with patients highlighting the value of its provision at the point of diagnosis, followed by an appointment where it was expected to be discussed. Patients reported that this allowed time to process the information and consider relevant questions. All participants felt the physiotherapist was an appropriate healthcare professional to be discussing decision-making about treatment with (Quote 3, Supplementary File 8). One patient reflected on the importance of the healthcare professional to be interested in the area and not “just going through the motions” [0012, patient], with another commenting on their preference for delivery by a “knee specialist” [0009, patient]. One participant suggested a one-to-one appointment may not be necessary. They felt the SDM intervention contained sufficient detail to support decision-making about treatment, particularly where ACLR was seen to be inevitable for young patients looking to return to high level cutting and pivoting sports.

Physiotherapists reported the intervention to be acceptable describing it to be non-burdensome, compatible with usual practice, low risk and within the scope of a qualified Physiotherapists’ skillset. The intervention was low/minimal cost to participants apart from the time taken to engage with it which was reported to be acceptable. One therapist reflected upon the increased time of a 60-minute appointment allowing for more in-depth conversations, which was preferred to 30-minutes. In contrast, some physiotherapists felt the intervention supported a reduction in consultation time.

Theme 2: Indicators of Effectiveness

The SDM intervention was reported to add value in several areas of practice by both patients and physiotherapists. Patients described the intervention to increase their knowledge of the injury and the treatment options available to them which supported them to engage in decision-making (Quote 4, Supplementary File 8). In contrast, one participant reflected on the lack of compelling evidence available and reported in the SDM intervention and suggested this added difficulty to the decision-making process (Quote 5, Supplementary File 8). Inclusion of the statistical data was seen to add value. It was viewed as evidence-based and relevant to what happens in real life. Several patients commented on use of statistics to support an individual assessment of the pros and cons of treatment, in line with what was important to them (Quote 6, Supplementary File 8). Physiotherapists also reported the level of detail, statistical data and information included in the intervention to be appropriate, relevant and useful. They reflected that, without the intervention, they would not have been able to offer the same level of evidence-based information as keeping up-to-date with the literature is challenging in the current NHS climate (Quote 7, Supplementary File 8).

The intervention was also described to support communication between patients and physiotherapists. Further, all patients reported to use the intervention outside of the trial, with friends, family members, healthcare professionals and employers to help communicate their injury and gain support to manage its consequences. Both physiotherapists and patients deemed the intervention to be trustworthy as it was derived from research and included references to the literature that were easy to locate should participants want to seek further information. As a result, some patients suggested to have not needed to conduct internet research to understand their injury further, saving them time from reading (or watching) unwanted levels of detail (Quote 8, Supplementary File 8). Physiotherapists further reported the intervention to have supported "buy in" from patients, who were perceived to have increased knowledge of their injury and treatment options available to them. They felt the intervention offered a good foundation level of knowledge for patients which supported a more pragmatic appointment that was structured, streamlined and tailored to each individual (Quote 9, Supplementary File 8). This was also mirrored by patients who reported the interventions value in providing them with access to healthcare language. Patients reflected on their 'non-medical background' and explained that the intervention increased their understanding of appropriate terminology, allowing them to have individualised conversations with healthcare professionals and ask important questions relevant to them (Quote 10, Supplementary File 8).

In addition, physiotherapists described the role of the intervention in empowering patients to be involved in their care. They felt this enabled a greater sense of control and increased patients' motivation as they were perceived to have higher accountability for the treatment and its outcomes. They reflected on the interventions presentation of information and felt it was fair and balanced towards both treatment options, ensuring it was effective for use with all patients following an ACL rupture. Physiotherapists further described the interventions utility in supporting their skills to deliver evidence-based information and have shared decision-making conversations with patients. This was reflected by physiotherapists in relation to patients following an ACL rupture, but also for patients with other musculoskeletal/orthopaedic conditions as part of usual practice as opposed to RCT type practice. They explained to feel more confident to take part in SDM and explore patient views about treatment (Quote 11, Supplementary File 8).

Theme 3: Trial Procedures

Both patients and physiotherapists had a positive attitude towards participating in the feasibility study. Patients reflected on the low burden of engagement with the intervention, completing baseline data collection and PROs electronically in-person or via mobile and email links. Three areas identified by physiotherapists for future trial refinement included, use of drop-in sessions for training refreshers (although not favoured by all), a QR code to access the clinician resources page on the website (replacing access via a password which was or could be forgotten) and a reminder/checklist of key intervention components to be embedded within a patient's electronic record. Physiotherapists reported the use of a 'study champion' and availability to email and receive a prompt response from a member of the research team to have been successful during the feasibility study and should be incorporated into a future main trial to support delivery.

Future contamination factors were also explored with physiotherapists. When asked about delivery of usual care in parallel to the SDM intervention (as part of an effectiveness trial), they expressed perceived difficulty with doing so given the new knowledge obtained from the SDM intervention. They favoured a design where treatment would be delivered for one intervention arm only. This is an important consideration and reflection given some therapists had also already begun using the intervention, or elements of it, in their usual practice.'

Discussion

- The subheadings make it much easier to follow but make sure to be consistent with heading vs subheadings formatting – you may need more subheadings following the feasibility headings or 'indicators of effectiveness' is much longer?
- At times some points are not specific enough. E.g. 'between 94-100%' may need clarification of what this means or removed.
- There are some good points in the discussion, but the structure still needs to be refined.

Thank you for the constructive comments. In response, alongside feedback from reviewer 1, we have made further revisions aimed at improving clarity and conciseness. We have amended the feasibility subheadings to match the exact wording used in the results section, ensuring consistency across the manuscript. Whilst the 'indicators of effectiveness' section remains longer, this reflects the discussion of both the satisfaction with decision scale results and qualitative exploration, which we feel are important to integrate for a comprehensive discussion. To clarify this, subheadings have been amended to include this detail. We have also reviewed and refined specific wording (e.g. clarifying ranges such as 94-100%) and revised the discussion to improve readability and flow. This has included removing over 25% of the overall wordcount. The revised discussion now reads:

'**DISCUSSION**
The results of this study confirm that the intervention is feasible to deliver and acceptable to both patients and physiotherapists. All four feasibility outcomes were achieved, with success rates ranging from 94 to 100% across the outcomes. Indicators of effectiveness suggest the interventions potential utility in supporting SDM processes for treatment decision-making following an ACL rupture. A full trial exploring the effectiveness of the SDM intervention is feasible.

Feasibility and acceptability
Recruitment rate
The overall recruitment rate of 95.2% was achieved ahead of schedule within four-and-a-half-months. This is encouraging and suggests patients are willing to consent to participate in a trial of this nature. During qualitative interviews, patients and physiotherapists reflected on the low burden of the intervention, timing at which it was delivered and appropriateness for a wide scope of patients following ACL rupture as eligibility criteria allowed for inclusion of participants with both acute and chronic ACL ruptures aged 18 and older. These factors likely aided recruitment and so a similar strategy in a future trial is warranted. It is however, important to highlight the high recruitment rate in the context of other musculoskeletal and orthopaedic research which is typically less than 50% ([19,20]).

Fidelity to the intervention

Fidelity of intervention delivery was 100% for all 11 physiotherapists involved in the study. Ahead of a participants' appointment, physiotherapists' were sent an email reminder of key procedures, likely contributing to high fidelity. Fidelity was assessed via a self-reported checklist by the treating physiotherapist; however, this was not externally verified for accuracy. Observation of consultations in a future study would offer clarity regarding fidelity of delivery.

Acceptability of the intervention

Overall acceptability was 93.67% across the 15 items in the acceptability questionnaire. A recent study exploring patient acceptability of a decision aid following Achilles tendon rupture (n=13) reported similar positive responses amongst patients regarding acceptability [21]. This suggests patients following an MSK/orthopaedic injury respond well to interventions supporting SDM and demonstrates appetite for future research in this area. A key perquisite to decision-making, and essential to SDM, is to ensure that patients are aware of the treatment options available to them [22]. In this study, 100% of patients felt 'the presentation of information was 'balanced' and fair towards each treatment option'. However, the lowest scoring question at 80% was that 'the presentation of information seemed in favour of having surgery' which conflicts the previous response. Interviews with patients revealed that although participants viewed the wording to be balanced, they felt research available to support a decision to proceed non-operatively was lacking. In addition, some participants described consultations which lacked clinical equipoise leaving patients uncertain about the validity of non-operative treatment. Similar to the previously discussed point, observation of consultations in a future trial may offer greater depth to the presentation of equipoise and impacts upon SDM.

Follow-up questionnaire completion

Follow-up outcome measure completion was 100% with acceptability of online methods via text and email links. Participants received a £20 voucher of their choice on receipt of questionnaire responses which may have contributed to the high response rate. During patient interviews, participants stated that the questionnaires were not burdensome, were relevant and easy to complete following the hyperlinks sent via text or email.

Indicators of Effectiveness and Qualitative Exploration

Although it was not the focus of the feasibility study to determine effectiveness (nor was it powered to do so), the SWD scale was used to explore indicators of the intervention's effect on decision-making processes. The average SWD score was 24.85 (95% CI 23.06-26.64, SD 3.82). Only one participant 'disagreed' with one statement regarding being adequately informed but did not respond to an interview invitation, limiting further exploration. Interviews with another participant, who indicated to 'neither agree nor disagree' with the same statement suggested this was due to limited information available in the literature about the non-operative treatment pathway (as discussed above). As this study was with a small number of participants, this will be an important avenue to consider in a future larger trial to understand potential unmet needs.

Patients reflected on the interventions utility in providing them with access to healthcare language and the subsequent ability to ask questions deemed relevant to them. NHS England outline current literacy levels in the UK as a crisis impacting upon people's health [23]. In England, a 2018 survey revealed 16.4% of the population to have poor literacy skills, defined as reading at or below the level of a 9-year-old [24]. A further 43% are thought to not understand written health information, with this rising to 60% when numbers and statistics are included [23,25]. The inclusion of statistical data was viewed

favourably by both patients and physiotherapists and there was no significant difference in outcomes for those identified to be at risk of poor health literacy. Assessing health literacy is an important element of a future trial to further understand the utility of the intervention in those with lower health literacy and levels of education.

There was some evidence of uncertainty with decision-making about treatment. One participant reported difficulty with the lack of clarity offered by the statistical data included in the intervention and discussed the challenge of making an informed decision. Uncertainty with decision-making in usual care following an ACL rupture has previously been reported, in addition to uncertainty with the non-operative treatment pathway [6, 26]. It is unknown how the participant in this feasibility study would have experienced the usual care pathway and whether the intervention had no effect on, introduced, or added to existing uncertainty. Evidence from other patients suggested that the intervention did not introduce doubt, but instead offered transparency to the treatment pathway which was reflected upon positively. Although previous research has identified most patients value the opportunity to be involved in decision-making, some may require more support and greater time for this process than others [27,28]. The patient demonstrating uncertainty with decision-making in this study, identified that they were likely to have responded differently (and more positively) to the follow-up questionnaires (acceptability and SWD) had they have been given more time to engage with the intervention before appraising it. Participation in decision-making has been associated with improved health outcomes and overall greater satisfaction with the consultation process [29,30]. Understanding change in SWD scores over time and in relation to specific points of interaction may therefore be important components of a future trial.

Most participants expected the outcomes of ACLR to be better than that detailed in the intervention. This expectation has previously been reported in an Australian cohort study, where only 24% of patients had returned to their pre-injury level of sport at 1-year despite 91% expecting to when asked preoperatively [31]. A 2023 predictors review, identified patients' positive estimation of their ability to return as a predictor of a successful return to physical activity [32]. However, it was highlighted that clinicians should take caution to ensure patients' expectations are not enhanced above levels that are realistic. This is an important role of the SDM intervention, with evidence from this study that engagement offered a pragmatic view of treatment outcomes that would have otherwise been assumed to be higher.

Clinician time constraints have previously been identified as a major barrier to implementing SDM [33]. A 2016 knee osteoarthritis SDM study reported no difference in overall consultation time when using an option grid compared to usual care [7]. One therapist with experience of a 30- and 60-minute consultation in the present study, acknowledged that whilst 60-minutes was preferred, delivery was achievable within 30 minutes. Reports from both patients and physiotherapists confirmed low burden of the intervention with no perceived negative effects to consultation time. This suggests that the intervention was appropriate for use during usual physiotherapy practice. One patient participant felt SDM was not prioritised by the physiotherapist due to time constraints. Exploration in a future full trial would confirm the interventions effectiveness in supporting SDM processes, how it is prioritised by clinicians, and its impact on consultation length.'

Strengths and Limitations

- I would suggest using the full term 'shared decision making' rather than 'SDM" here

Thank you for your suggestion. As per manuscript formatting guidelines, the term ‘shared decision-making’ was expanded at first use, and the abbreviation is used consistently thereafter. We believe this approach maintains readability whilst adhering to standard academic formatting.

- Some of the point could be more concise. For example. ‘Qualitative interviews with patients and clinicians provided deeper exploration of feasibility, acceptability and indicators of effect of the intervention’

The text has been amended for clarity and now reads:
‘Strengths, Limitations and Reflexive Considerations
Data were collected on participants’ ethnicity, recognised in the 2022 (most recent) UK NLR report as a future priority to support understanding of the epidemiology and outcomes following ACL rupture [34]. Whilst we acknowledge the present study included only 20 participants in one area of the UK, capturing this data may support future understanding of the demographics of those with ACL ruptures. Without consistent reporting of ethnic background in the literature, it is unclear whether the findings of this study are transferable to the wider UK ACL rupture population. The population included those from a wide age range, with varying levels of pre-injury and current physical activity, who sustained their ACL rupture via contact and non-contact mechanisms. These demographics would suggest that the study captured an appropriate sample encompassing a range of characteristics representative of the reported adult ACL population in the current literature [9].

A second strength of this study is that participants’ level of deprivation, level of education and health literacy were recorded, to support understanding of the SDM interventions utility in those at risk of poorer healthcare experiences and outcomes. Whilst no significant differences were detected between groups, this only offers an indication of the intervention’s utility in SDM process as the study was not powered to detect effectiveness. This is an important area of exploration in a future trial to ensure individuals from lower socioeconomic backgrounds are not at risk of marginalisation from SDM processes. All participants identified to be at risk of poor health literacy declined to be contacted to share their experiences in a qualitative interview. We therefore may not have captured a full range of perceptions and experiences, particularly of those at risk of poor health literacy.

Although there were provisions in place to recruit non-English speaking participants, none were identified as eligible for the trial. Future consideration to understand how the material may translate into other languages and its utility with non-English speaking participants may therefore be needed.

Further, the qualitative interviews were carried out by the lead researcher who also identifies as a clinician at one of the physiotherapy departments running the study and who has previously practiced at the second department. The lead researcher has been a member of the clinical team for several years. This may have limited the discussions with physiotherapists who may not have felt comfortable accounting negative experiences to a clinical colleague.’

Conclusion:

- Between 94-100% of what?
- The conclusion could still better match the objectives and focus on what the main findings.

- E.g. Indicators of effectiveness showed the shared decision making tool… based on…
- Maybe finish with a stronger statement on why shared decision making is needed in ACL decision making and the next step identified/planned?

Thank you for these suggestions. We have revised the conclusion to provide further clarity, more clearly align with the study objectives and to emphasise key findings. We have also included a summary statement on the importance of SDM in ACL decision making and proposed next steps. The conclusion now reads: 'All four feasibility outcomes were achieved, with success rates ranging from 94 to 100% across the outcomes. This study has demonstrated that implementation of an SDM intervention in the ACL rupture pathway is acceptable to both patients and physiotherapists and can be delivered with high fidelity. Exploring indicators of effectiveness, through quantitative and qualitative data, identified the interventions utility in improving patient knowledge, supporting patients to understand medical terminology to formulate clinically relevant questions, facilitating SDM conversations, empowering patients to be involved in decision-making about their treatment and providing up-to-date clinically relevant information for both patients and clinicians. These findings support the need for further evaluation in a definitive trial, given the importance of SDM in ensuring patient-centred care in ACL injury management, as evidence to date highlights that surgical intervention is not necessary for all.'
